# COVID-19 Risk Assessment for the Tokyo Olympic Games

**DOI:** 10.3389/fpubh.2021.730611

**Published:** 2021-10-25

**Authors:** Wenhui Zhu, Jie Feng, Cheng Li, Huimin Wang, Yang Zhong, Lijun Zhou, Xingyu Zhang, Tao Zhang

**Affiliations:** ^1^Department of Epidemiology and Health Statistics, West China School of Public Health and West China Fourth Hospital, Sichuan University, Chengdu, China; ^2^Sichuan Center for Disease Control and Prevention, Chengdu, China; ^3^Thomas E. Starzl Transplantation Institute, University of Pittsburgh Medical Center, Pittsburgh, PA, United States

**Keywords:** COVID-19, SEIARH model, interventions, Tokyo Olympic Games, risk assessment

## Abstract

**Introduction:** As of June 7, 2021, the outbreak of Coronavirus Disease 2019 (COVID-19) has spread to more than 200 countries. The global number of reported cases is more than 172.9 million, with more than 3.7 million deaths, and the number of infected individuals is still growing rapidly. Consequently, events and activities around the world were canceled or postponed, and the preparation for sporting events were greatly challenged. Under such circumstances, about 11,000 athletes from ~206 countries are arriving in Tokyo for the 32nd Summer Olympic Games. Therefore, it is urgently necessary to assess the occurrence and spread risk of COVID-19 for the Games.

**Objectives:** To explore effective prevention and control measures for COVID-19 in large international events through simulations of different interventions according to risk assessment.

**Methods:** We used a random model to calculate the number of initial infected patients and used Poisson distribution to determine the number of initial infected patients based on the number of countries involved. Furthermore, to simulate the COVID-19 transmission, the susceptible-exposed-symptomatic-asymptomatic-recovered-hospitalized (SEIARH) model was established based on the susceptible-exposed-infectious-recovered (SEIR) mathematical model of epidemic diseases. According to risk assessment indicators produced by different scenarios of the simulated interventions, the risk of COVID-19 transmission in Tokyo Olympic Games was assessed.

**Results:** The current COVID-19 prevention measures proposed by the Japan Olympic Committee need to be enhanced. And large-scale vaccination will effectively control the spread of COVID-19. When the protective efficacy of vaccines is 78.1% or 89.8%, and if the vaccination rate of athletes reaches 80%, an epidemic prevention barrier can be established.

## Introduction

The 32nd Summer Olympic Games (Games of the XXII Olympiad) will be held from July 23 to August 8, 2021 in Tokyo, Japan (1). However, athletes and spectators of the Olympics Games are easily exposed to the Coronavirus Disease 2019 (COVID-19). As of June 7, 2021, the outbreak of COVID-19 has spread to more than 200 countries (2). The global epidemic situation remains grim, with more than 172.9 million global cumulative reported cases and more than 3.7 million deaths (3), while the number of infected individuals is still growing rapidly. Consequently, numerous major events and activities around the world have been canceled or postponed, and preparations for all parts of upcoming sport events were greatly challenged. Under such circumstances, about 11,000 athletes from ~206 countries and regions are arriving in Tokyo for the 32nd Summer Olympic during the summer of 2021 (4), along with coaches, referees, and associated International Sport Organization officials, which will definitely increase the possibility of infectious disease outbreaks and transmissions. Although the International Olympic Committee (IOC) has stated that overseas audiences will not be allowed to enter Japan to view the Tokyo Olympic Games (5), it is far from enough to ensure the safety of the Olympic Games in terms of COVID-19. Therefore, it is urgently necessary to commence an evaluation for the risk of COVID-19 under different prevention measures.

So far the COVID-19 prevention measures proposed by the Japan Olympic Committee (JOC) include the following aspects (6, 7):

1. Keep a minimum of two meters from athletes at all times. Keep a minimum of one meter from others. All Games participants must minimize contact within one meter of Games participants who have already been in Japan for more than 14 days, and Japanese residents.
2. During your stay in Japan, you will be expected to limit your activities to what is required to carry out your role.
3. All participants are required to take two COVID-19 tests before their flight to Japan. All other Games participants will be tested daily for three days after their arrival. After the first 3 days and throughout their stay, they will be tested regularly, based on the operational nature of their role and level of contact with athletes.

Thomas Bach, the president of IOC, said that if a vaccine becomes available in time for the July 23–August 8 Games in 2021, the IOC would foot the bill (8). However, since vaccination is voluntary for athletes, different vaccine coverage may produce different effects on an outbreak in the Olympic Village. In light of how this has not been studied so far, we used different vaccine coverage statistics to simulate the transmission of COVID-19 in this study.

**Figure 1:**
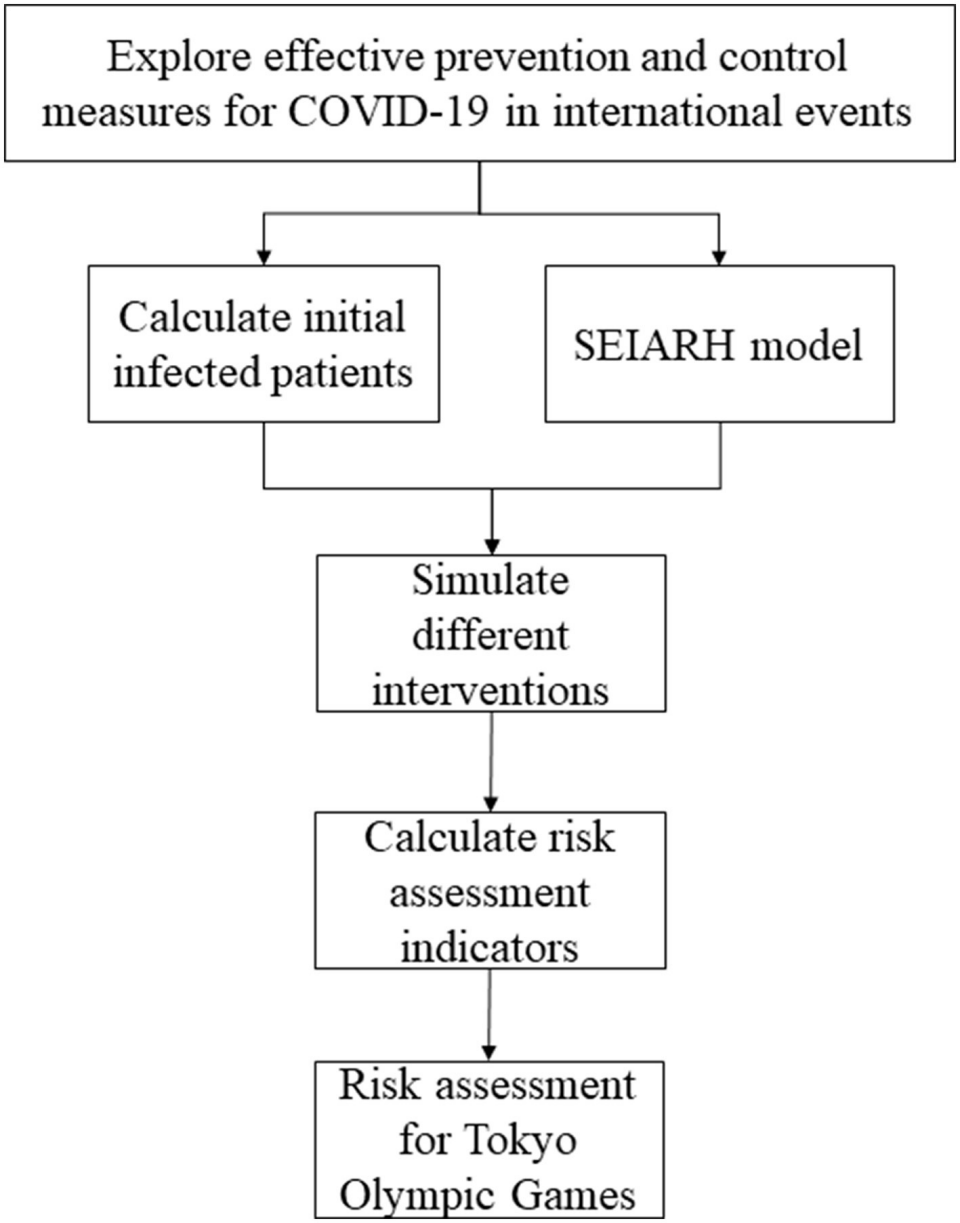
The procedure of analysis.

In this study, to assess the risk of COVID-19 during the Tokyo Olympic Games, a simulation study based on the transmission dynamic model was carried out. Firstly, we collected the number of athletes from different countries participating in the Tokyo Olympic Games, the current COVID-19 infection probability of each country, and the transmission parameters of the COVID-19 model. Secondly, utilizing the initial number of asymptomatic infections, the number of contacts and other aspects, the susceptible-exposed-symptomatic-asymptomatic-recovered-hospitalized (SEIARH) model was established. Thirdly, in order to carry out risk assessments, the secondary infectors on the 17th day and peak hour of onset were calculated. Through realizing a comparison of the expected risks of COVID-19 under different prevention strategies, this study provided quantitative reference evidence regarding the formulation of COVID-19 prevention and control programs for the Tokyo Olympic Games. The specific process of analysis is shown in Figure 1.

## Materials and Methods

The methods of this study consisted of three parts. Firstly, to determine the number of initial infected patients based on the number of countries coming to Japan, Poisson distribution was used. Secondly, based on the COVID-19 transmission mechanism, the SEIARH model was established to simulate COVID-19 transmission. And then we analyzed the robustness of the SEIARH model. Thirdly, according to the results of the SEIARH model, the risk assessment indicators (secondary infectors on the 17th day, peak hour of onset/d) were calculated.

### Data Collection and Preparation

National infection probability π_*i*_(*i* = 1, 2, ⋯ , *n*) was approximated according to public data (3):


(1) $$\pi_{i}=\frac{I_{i}}{N_{C\_i}},\;i=1,2,\cdots ,n$$

where *I*_*i*_ is the number of infections in country *i*; *N*_*C*_*i*_ is the total population number of country *i*.

As the Olympic Games are held every 4 years, little change existed in the total number of the adjacent two sessions (9). Besides, the number of athletes was not available before the announcement of IOC, so we used the number of athletes announced in the last Olympic Games as a reference (10). Furthermore, we set the initial asymptomatic infected population *A* (0) as 10 because the previous imported asymptomatic infected cases did not exceed 10 at one time.

For the parameters of the SEIARH model, we referred to the published COVID-19 classic retrospective study (11–18).

### Determining the Number of Initial Infected Patients

We assumed the presence of COVID-19 infectors at entry who were not identified by entry quarantine or health check-up. Here we leveraged the probability of infection π_*i*_ to approximate the number of overseas import infectors. Furthermore, since the number of participants and the spread of COVID-19 vary across different countries, the initial infected patients referred to unidentified infectors among athletes, coaches, referees, officials, and others who entered Japan for the Tokyo Olympics Games.

The number of infected patients assumed from different regions *i*(*i* = 1, 2, ⋯ , *n*) is *X*_*i*_. It can be approximately seen that the Poisson distribution of compliance parameter λ_*i*_ = *N*_*i*_π_*i*_, which is in the form of:


(2) $$P\left\{X_{i}=k\right\}=\frac{{\text{λ}}_{i}^{k}}{k!}e^{-{\text{λ}}_{i}},i=1,2,\cdots ,n;k=0,1,\cdots ,$$

where *N*_*i*_ is the number of athletes, coaches, referees, and officials from different regions; π_*i*_ is the infection probability of immigrants from different regions. The infected input probability *P*_*i*_*imported*_ of a person from region *i* was:


(3) $$P_{i\_imported}=1-P\left\{X_{i}=0\right\},i=1,2,\cdots n.$$

Furthermore, the total number of initial infected patients could be represented as:


(4) $$I_{imported}=\sum_{i=1}^{n}N_{i}P_{i\_imported}.$$

### Simulating the Transmission of COVID-19 During the Tokyo Olympic Games

**Figure 2:**
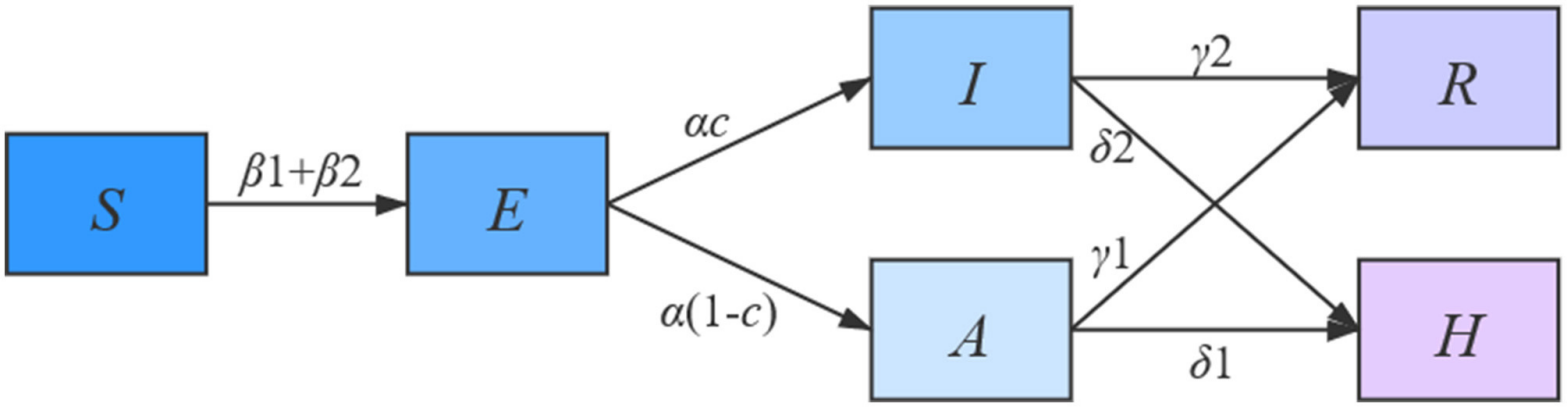
Flow diagram of the SEIARH model.

The mathematical models of infectious diseases can be classified into two types by the level of data unit, i.e., the micro-dynamic and macro-dynamic models. The former type of model includes scale-free networks, small-world networks, and so on (19, 20), aiming to show the transmission process of the disease based on the individual level. Thus, it requires a large amount of high-quality personal data. However, since the purpose of this study was to simulate the transmission of COVID-19 on the population level, it was unnecessary and inefficient to collect personal data for model building. Hence, this made the micro-dynamic model unsuitable for our simulation study. On the contrary, the macro-dynamic model provides us with tools for monitoring population flow among different health statuses. For example, the traditional susceptible-exposed-infected-recovered (SEIR) model classifies the population into four categories by its name (21–24). However, for the specific purpose of simulating COVID-19 transmission in this study, neither the difference between asymptomatic and symptomatic patients nor the requirement of isolation treatment was reflected by the SEIR model. To this end, we added both the A (asymptomatic) and H (hospitalized) components to the SEIR model to establish the SEIARH model for the simulation of COVID-19 transmission. The transmission process is shown in Figure 2.

For the simulation study, the discrete-time stochastic compartment model for COVID-19 infection was constructed as:


(5) $$\{\;\begin{array}{l} \frac{dS}{dt}=-\frac{r_{1}(t)\beta_{1}\left(I+A\right)S}{N}-\frac{r_{2}(t)\beta_{2}ES}{N}\\ \frac{dE}{dt}=\frac{r_{1}(t)\beta_{1}\left(I+A\right)S}{N}-\alpha E+\frac{r_{2}(t)\beta_{2}ES}{N}\\ \frac{dI}{dt}=\alpha cE-\gamma_{1}I-\delta_{1}I\\ \frac{dA}{dt}=\alpha \left(1-c\right)E-\gamma_{2}A-\delta_{2}A\\ \frac{dH}{dt}=\delta_{1}I+\delta_{2}A-\gamma_{3}H\\ \frac{dR}{dt}=\gamma_{1}I+\gamma_{2}A+\gamma_{3}H \end{array}$$

The parameters are defined in Table 1. As the Japan authority has proposed that “During your stay in Japan, you will be expected to limit your activities to what is required to carry out your role” (6). Therefore, the range of activities of athletes outside the Olympic Village was not considered, and it was reasonable to set the total population number *N* = 11,000. Based on the historical data of Wuhan, China between January 11 and March 13, we used the Markov Chain Monte Carlo Method (MCMC) to estimate the recovery rate of symptomatic infected individuals. It turned out that the results of data-driven methods were consistent with the real-world results (25), where the 95% *CI* of γ_1_ and γ_2_ was (0.0085, 0.0085) and (0.0085, 0.0085), respectively. More details can be found in Supplementary Material A. In addition, we also initialized the other simulation parameters in Table 1 through literature review (11–18).

**Table 1:** Parameter definition and estimation.

**Parameter**	**Definition**	**Value**	**References**
*N*	The total population number	1.1*10^4^	(4)
β_1_	Probability of transmission from infected individuals to susceptible individuals	0.15747	(11)
β_2_	Probability of transmission from exposed individuals to susceptible individuals	0.78735	(11)
*r*_1_(*t*)	Average number of infected class contact with susceptible class	2.20	(12)
*r*_2_(*t*)	Average number of infected class contact with exposed class	2.22	(13)
α	Probability of susceptible individuals becoming infected individuals	1/5.2	(14)
δ_1_	Transition rate of symptomatic infected individuals to the quarantined infected class	0.6	(15)
δ_2_	Transition rate of asymptomatic infected individuals to the quarantined infected class	0.4	(15)
γ_1_	Recovery rate of symptomatic infected individuals	0.0085	MCMC
γ_2_	Recovery rate of asymptomatic infected individuals	0.0085	MCMC
γ_3_	Recovery rate of hospitalized individuals	0.15	(17)
*c*	Probability of infected class have symptoms	0.4	(18)
*S* (0)	Initial susceptible population	1.1*10^4^	(4)
*E* (0)	Initial exposed population	0	
*A* (0)	Initial asymptomatic infected population	10	
*I* (0)	Initial symptomatic infected population	0	Assumed
*R* (0)	Initial recovered population	0	
*H* (0)	Initial quarantined infected population	0	

*This study mainly considered the effect of different measures on the prevention and control of the Tokyo Olympic Games in the case of missed detection of asymptomatic infectors, so the initial asymptomatic infectors were 10, and the remaining initial values were 0*.

Since the Tokyo Olympic Games lasted 17 days, a total of *t* = 1, 2, ⋯ , 17 data points were simulated. Figures 3–16 show the simulated results. The goal for this simulation was to investigate the transmission of COVID-19 during the Tokyo Olympic Games. According to the general principles of the simulation study design (26), we implemented our simulation study following the details as presented in Table 2.

**Figure 3:**
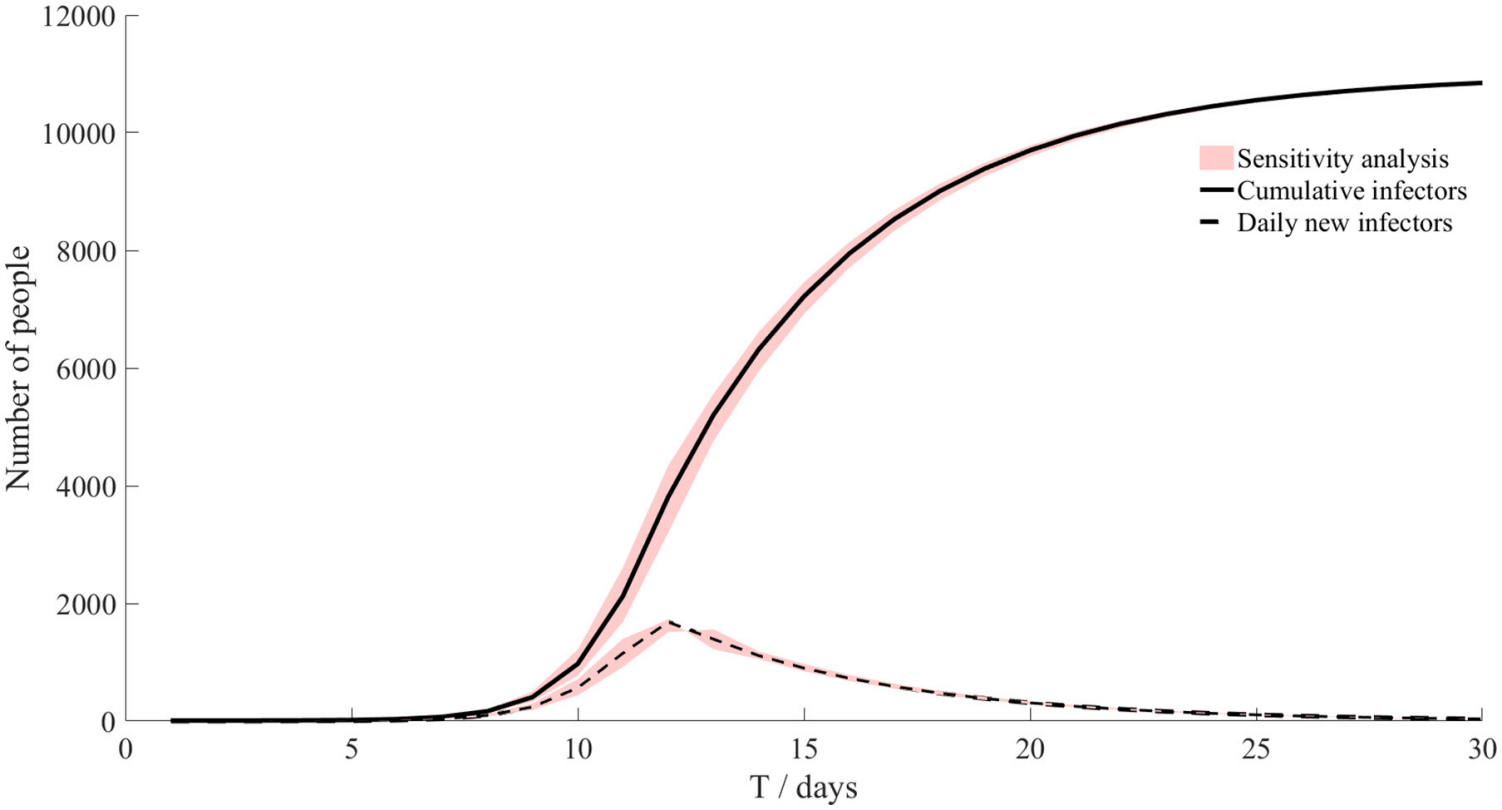
Transmission without intervention.

**Figure 4:**
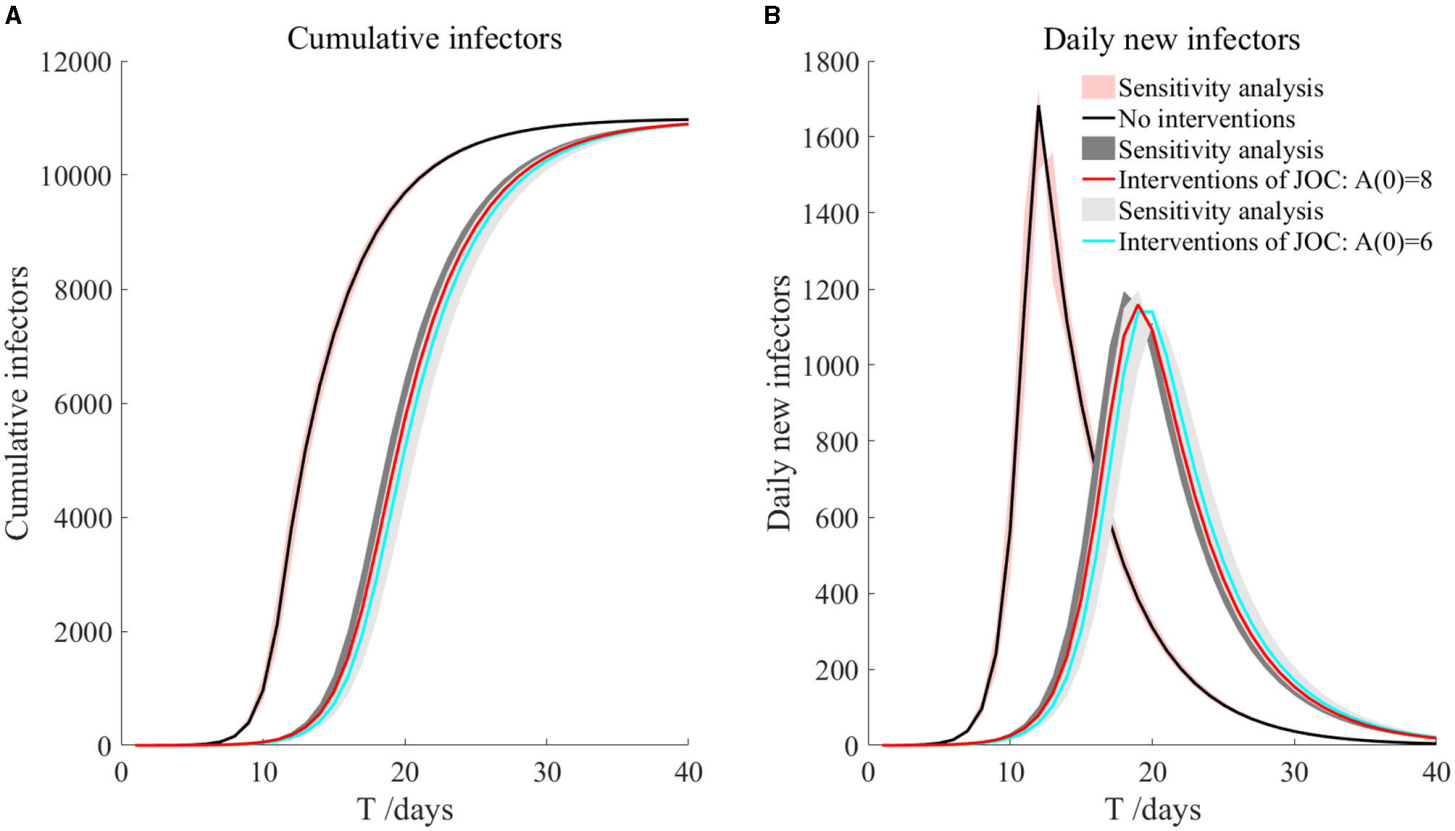
The transmission after JOC interventions.

**Figure 5:**
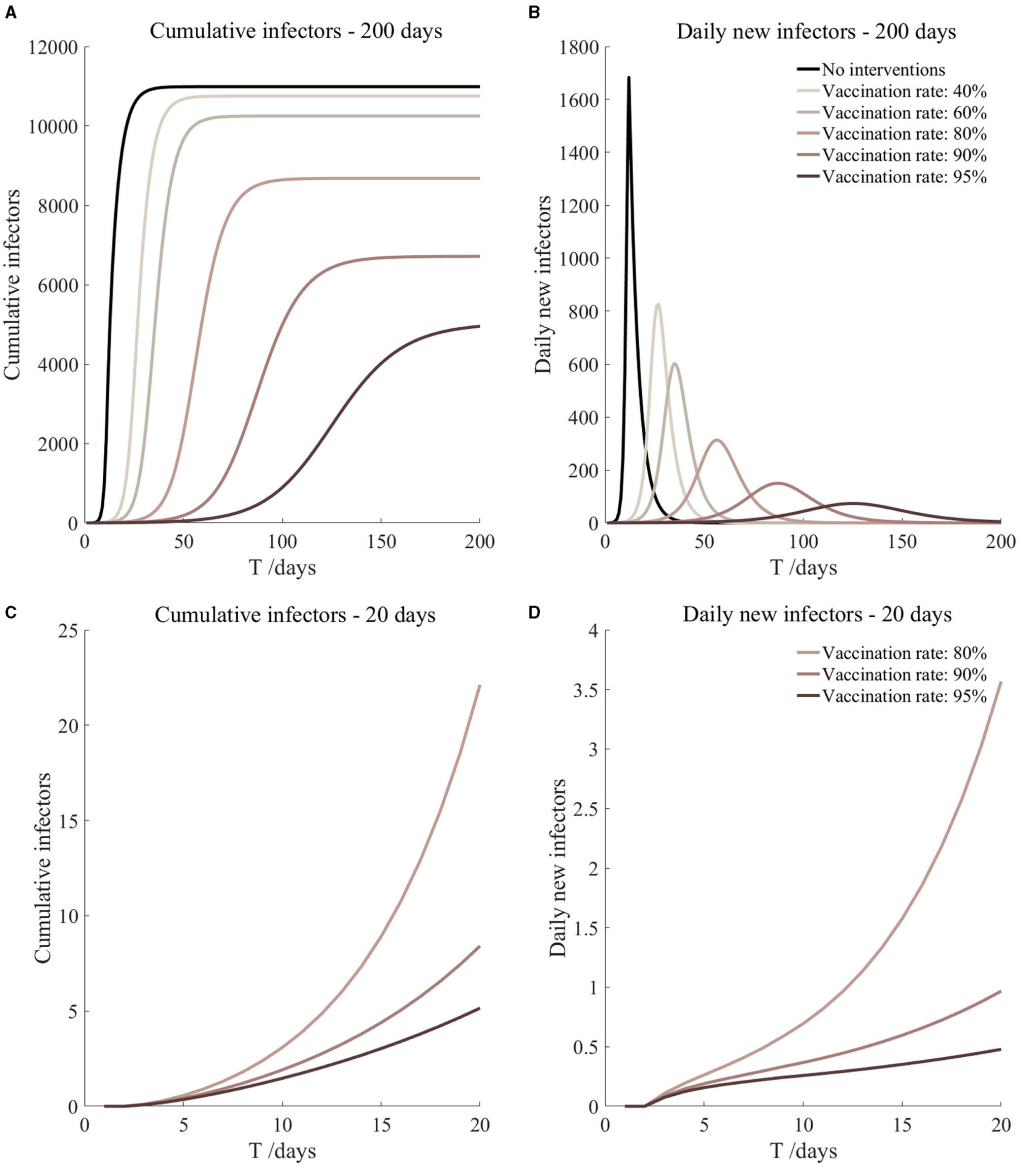
Vaccinated but not quarantined. Assume pre-flight screening can identify 20% of asymptomatic infectors.

**Figure 6:**
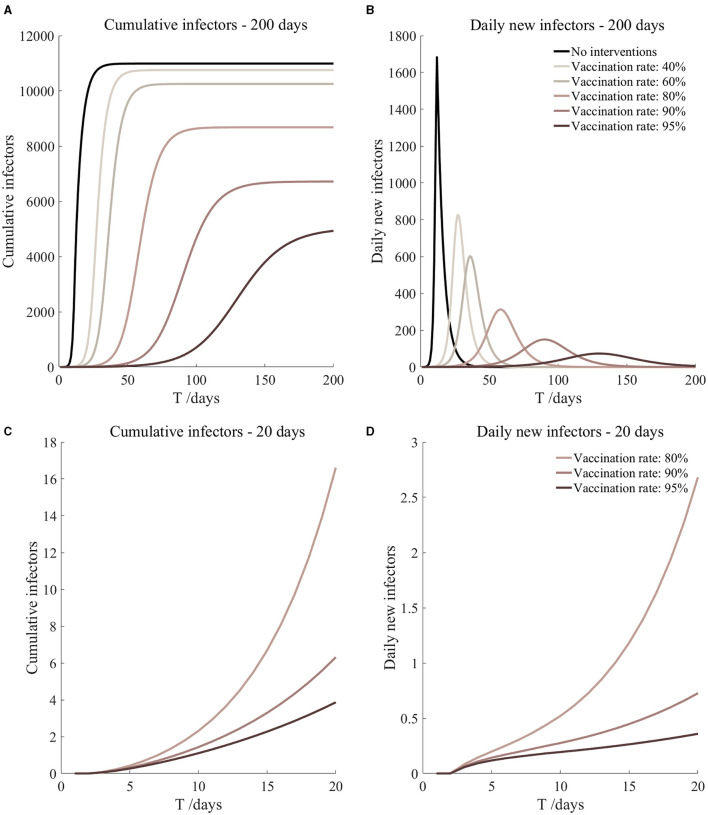
Vaccinated but not quarantined. Assume pre-flight screening can identify 40% of asymptomatic infectors.

**Figure 7:**
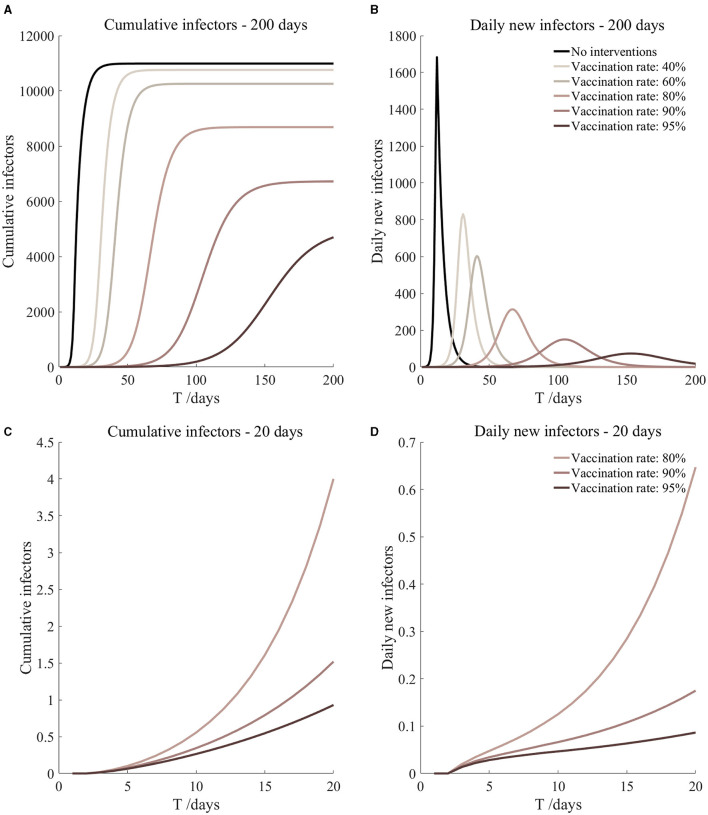
Vaccination and 7-day compulsory quarantine. Assume pre-flight screening can identify 20% of asymptomatic infectors, 7-day compulsory quarantine has about an 18% risk of missed detection.

**Figure 8:**
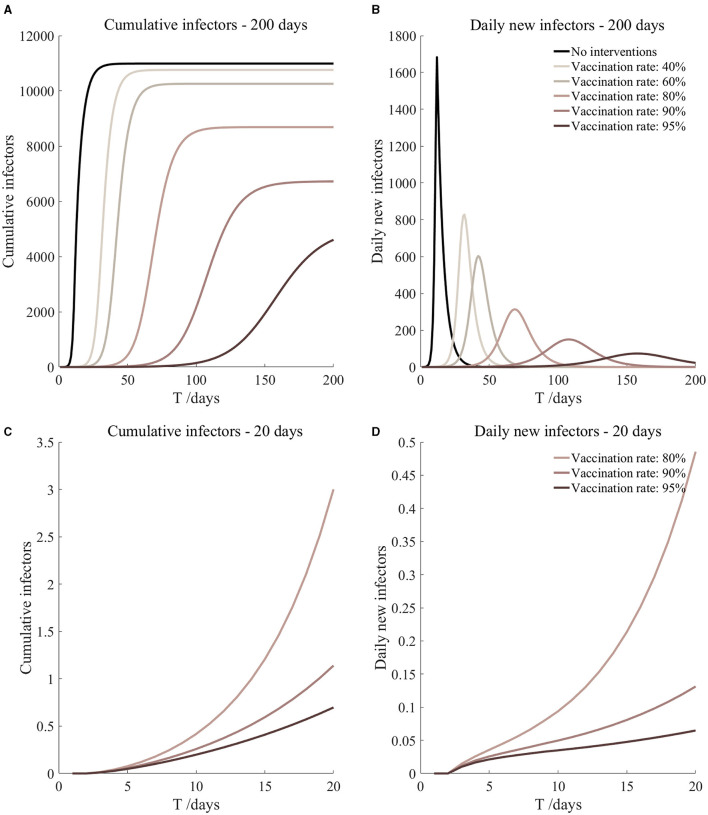
Vaccination and 7-day compulsory quarantine. Assume pre-flight screening can identify 40% of asymptomatic infectors, 7-day compulsory quarantine has about an 18% risk of missed detection.

**Figure 9:**
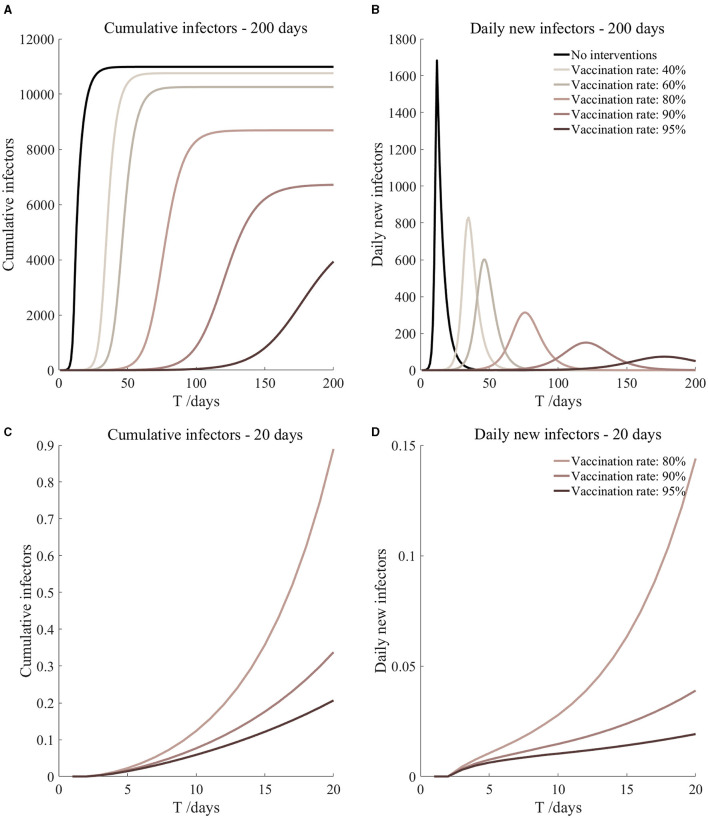
Vaccination and 14-day compulsory quarantine. Assume pre-flight screening can identify 20% of asymptomatic infectors, 14-day compulsory quarantine has about a 4% risk of missed detection.

**Figure 10:**
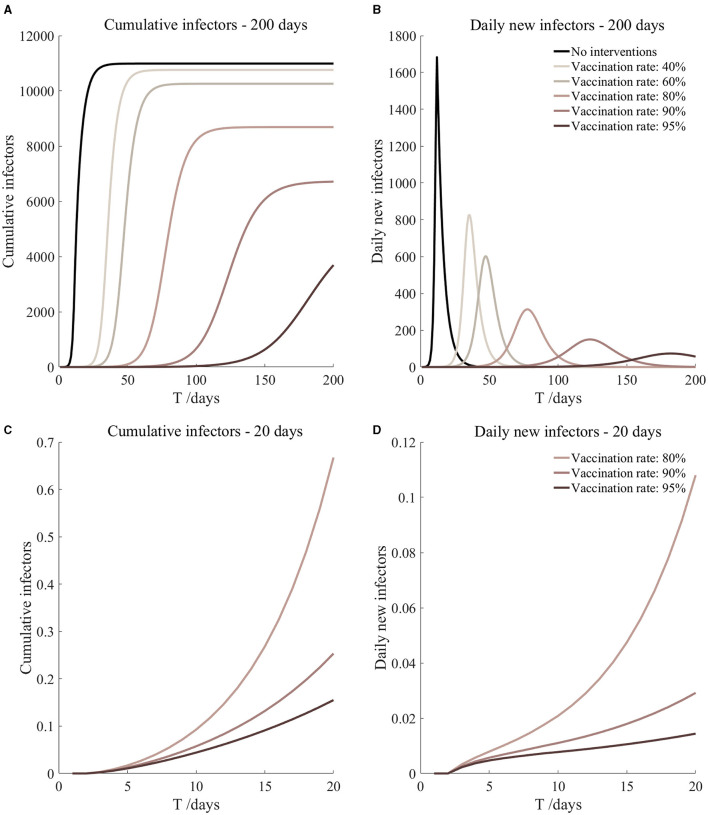
Vaccination and 14-day compulsory quarantine. Assume pre-flight screening can identify 40% of asymptomatic infectors, 14-day compulsory quarantine has about a 4% risk of missed detection.

**Figure 11:**
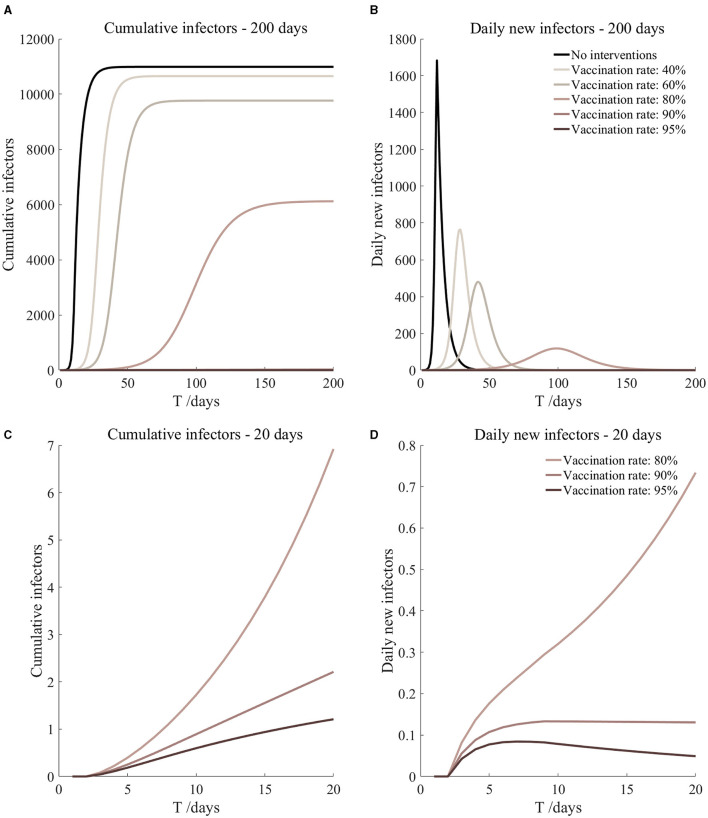
Vaccinated but not quarantined. Assume pre-flight screening can identify 20% of asymptomatic infectors.

**Figure 12:**
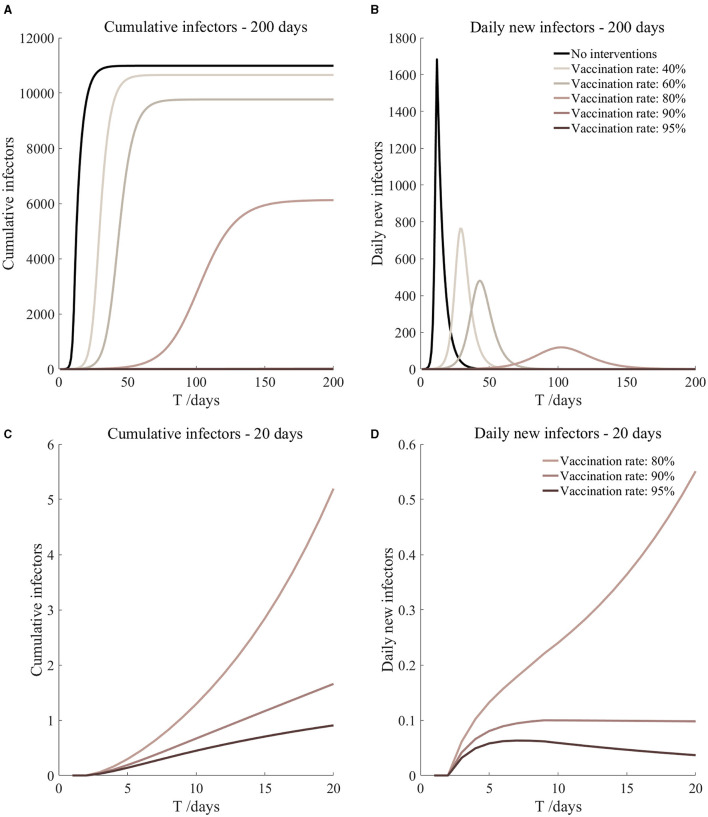
Vaccinated but not quarantined. Assume pre-flight screening can identify 40% of asymptomatic infectors.

**Figure 13:**
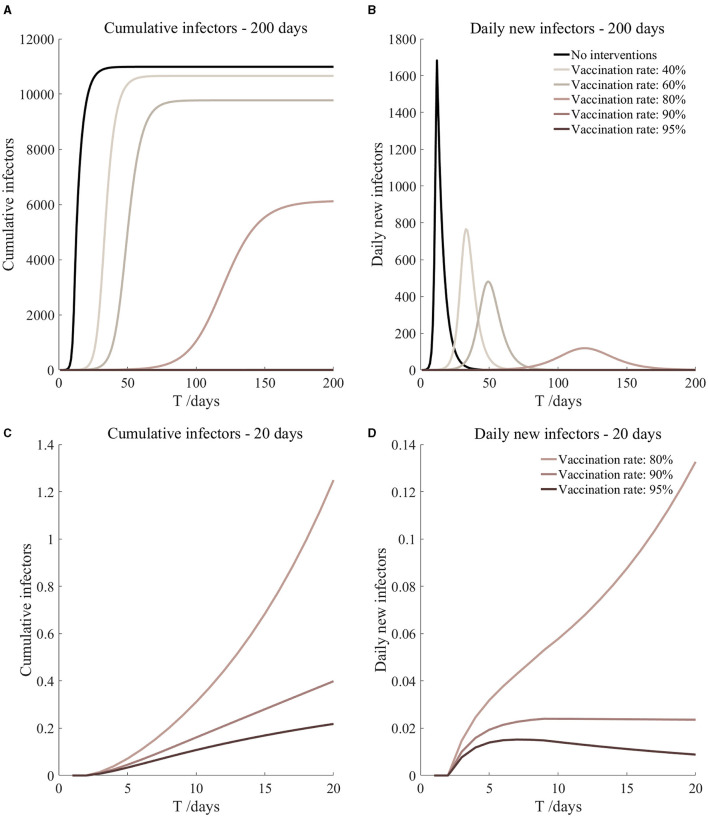
Vaccination and 7-day compulsory quarantine. Assume pre-flight screening can identify 20% of asymptomatic infectors, 7-day compulsory quarantine has about an 18% risk of missed detection.

**Figure 14:**
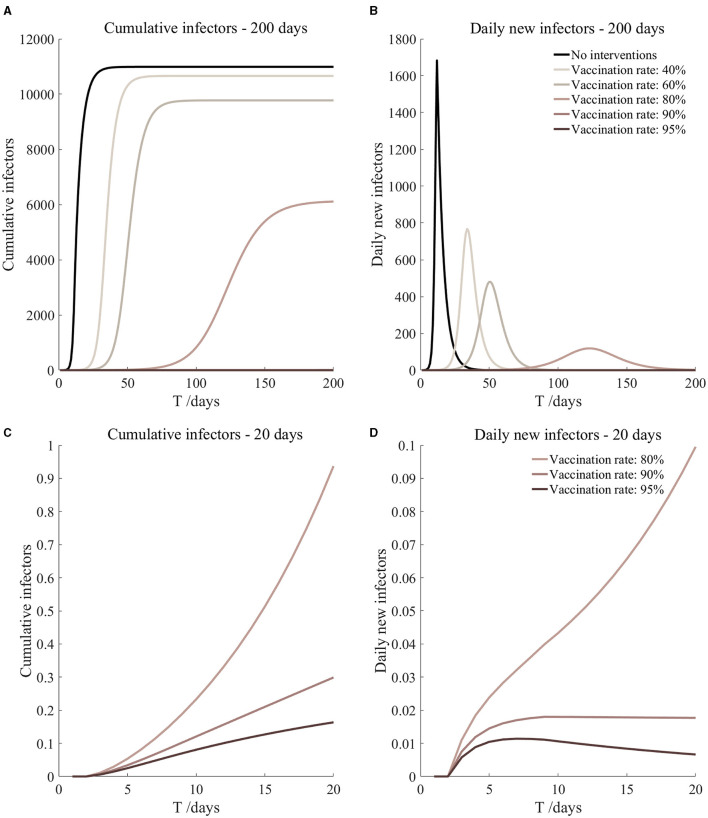
Vaccination and 7-day compulsory quarantine. Assume pre-flight screening can identify 40% of asymptomatic infectors, 7-day compulsory quarantine has about an 18% risk of missed detection.

**Figure 15:**
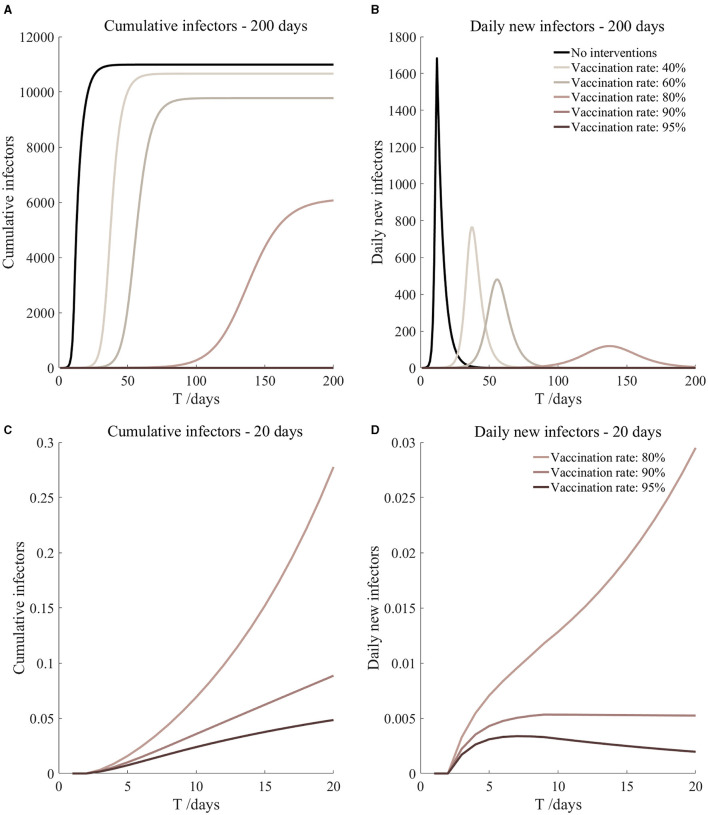
Vaccination and 14-day compulsory quarantine. Assume pre-flight screening can identify 20% of asymptomatic infectors, 14-day compulsory quarantine has about a 4% risk of missed detection.

**Figure 16:**
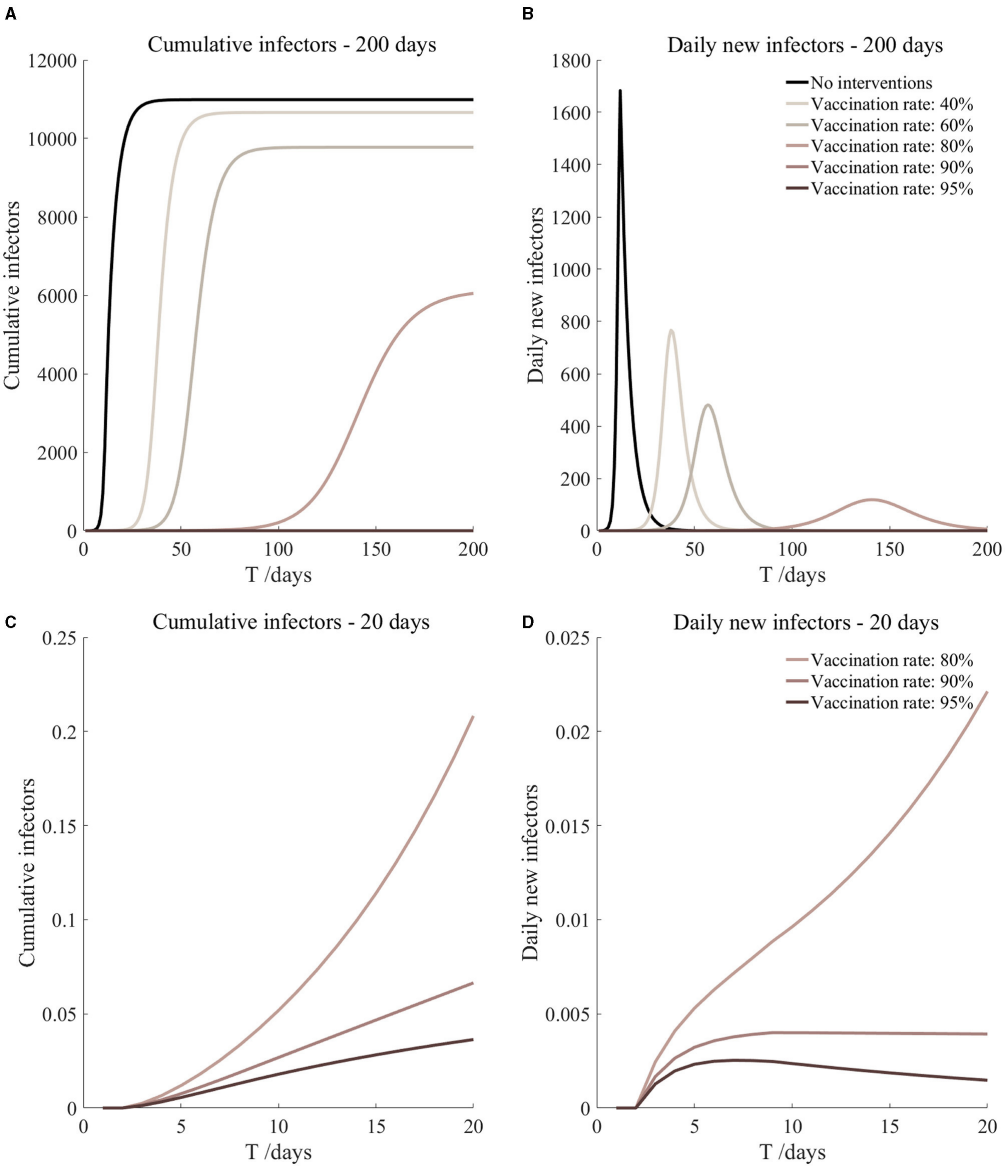
Vaccination and 14-day compulsory quarantine. Assume pre-flight screening can identify 40% of asymptomatic infectors, 14-day compulsory quarantine has about a 4% risk of missed detection.

**Table 2:** Key steps and results in the planning, coding, and analysis of this simulation study.

	**Section**
**Planning**	2
Aims	
• Simulate the transmission of COVID-19 during the Tokyo Olympic Games.	
Data-generating mechanisms	
• The parameters of the SEIARH model came from MCMC estimations based on real data or previous studies.	
Target of analysis	
• To explore effective prevention and control measures for COVID-19 in large international events.	
Methods	
• Used random model to calculate the number of initial infected patients and used Poisson distribution to determine the number of initial infected patients based on the number of countries involved. • Established the SEIARH model to simulate the transmission of COVID-19. • Estimated risk assessment indicators by different scenarios of the simulated interventions.	
Performance measures	
• Analyzed the robustness of the model by increasing or decreasing parameters β_1_ and β_2_ at the same time.	
**Coding and Execution**	2
• More details can be found in Supplementary Material B.	
**Analysis**	3
• The current COVID-19 prevention measures proposed by the Japan Olympic Committee need to be enhanced. • Large-scale vaccination will effectively control the spread of COVID-19. When the protective efficacy of vaccine is 78.1 or 89.8% and if the vaccination rate of athletes reaches 80%, an epidemic prevention barrier can be established.	

The current regulations required all participants to take two COVID-19 tests before their flight to Japan, and the number of contacts should be strictly controlled after entry (7, 27, 28). To make the experiment more realistic, we defined the number of susceptible individuals exposed by infected [*r*_1_(*t*)] and exposed [*r*_2_(*t*)] as piecewise functions of time *t*, where *t* is the number of days after entry. The function forms were given as follows.


(6) $$r_{1}(t)=\{\;\begin{array}{l} r_{10},\;0\leq t\leq 3\\ r_{11},\;3\leq t\leq 14\\ r_{12},\;14\leq t\leq 17\\ r_{13},\;17\leq t\leq 21 \end{array},\;r_{2}(t)=\{\;\begin{array}{l} r_{20},\;0\leq t\leq 3\\ r_{21},\;3\leq t\leq 14\\ r_{22},\;14\leq t\leq 17\\ r_{23},\;17\leq t\leq 21 \end{array}$$

### Sensitivity Analysis

In order to verify the robustness of the SEIARH model, we analyzed the sensitivity of the model. The parameter β_1_ and β_2_ were increased or decreased by 5% at the same time. These two parameters directly affect the final number of infectors. The cumulative infectors and daily new infectors were observed to determine whether the model was effective.

### Assessing the Risks of COVID-19 Under Different Prevention Measures

Based on the results of the SEIARH model, we calculated the risk assessment index of COVID-19: peak hour of onset and secondary infectors on the 17th day. Since the Tokyo Olympic Games lasted for 17 days, secondary infectors on the 17th day were used as indicators. Secondary infectors on the 17th day refers to the number of daily new infectors at the end of the Tokyo Olympic Games; hour of onset is the time *t* corresponding to the maximum number of daily new infectors.

## Results

### Transmission Simulation Without Intervention

In this case, we ignored the activities of athletes outside the Olympic Village, and then we used the parameters of the SEIARH model in Table 1 to simulate the transmission within the Olympic Village in Japan without intervention, as shown in Figure 3. The number of daily new secondary infectors reached the peak on the 12th day, which was 1,683. The cumulative infectors were in an S-shaped curve.

The red-shadowed region in Figure 3 is the result of sensitivity analysis. We found that the cumulative infectors and daily new infectors changed little, indicating that the model was robust.

### Transmission Simulation Under Current Interventions of the Japan Olympic Committee (JOC)

According to the current prevention and control measures of JOC, the parameters of the average number of infected class contact with susceptible class (*r*_1_), average number of infected class contact with exposed class (*r*_2_), initial asymptomatic infected population [*A*(0)] were adjusted, and the specific settings are shown in Table 3.

**Table 3:** Parameter adjustment and transmission simulation.

**Prevention and control measures**	**Parameter adjustment**	**Setting basis**	**Secondary infectors on the 17th day**	**Peak hour of onset/d**
No intervention (Figure 3)	–	–	587.8	12
Keep a minimum of two meters from athletes at all times. Keep a minimum of one meter from others. Take two COVID-19 tests before their flight to Japan (Figure 4)	*r*_1_ = 1.2, *r*_2_ = 1.22, *A*(0) = 8	Assume pre-flight screening can identify 20% of asymptomatic infectors	857.6	19
The same as above (Figure 4)	*r*_1_ = 1.2, *r*_2_ = 1.22, *A*(0) = 6	Assume pre-flight screening can identify 40% of asymptomatic infectors	729.7	21

As can be seen from Figure 4, based on the measures currently proposed by JOC, it was assumed that the screening before take-off can reduce 20 and 40% of asymptomatic infectors, respectively, but the prevention and control effect was not ideal. The total number of secondary infectors still reached the level of no intervention measures, but the duration increased; the peak value of daily new secondary infectors decreased, reaching 1,159 on the 19th day and 1,141 on the 20th day, respectively. Secondary infectors on the 17th day reached 857.6 and 729.7, respectively. Compared with no intervention measures, the situation of current interventions of JOC improved.

The shadowed region in Figure 4 is the result of sensitivity analysis. It can be seen that cumulative infectors and daily new infectors were almost unchanged, indicating that the model was robust. More importantly, through the simulations of the current prevention and control measures, it could be found that it was not enough to rely solely on the existing measures. Other measures (such as vaccination) must be supplemented to effectively reduce the risk of the epidemic during the Tokyo Olympic Games. We will discuss it further in the next section.

### Transmission Simulation Under Other Interventions

According to the simulation of intervention measures set in the above sections, we found that the current prevention and control effect was not ideal. But for various infectious diseases, vaccines are the most effective way to eradicate the transmission of infectious diseases. Therefore, based on the intervention measures proposed by JOC, we considered the transmission of athletes after vaccination. The transmission simulation was carried out by adding prevention and control scenarios such as “vaccinated but not quarantined,” “vaccination and 7-day compulsory quarantine,” and “vaccination and 14-day compulsory quarantine.” The protective efficacy of any vaccine cannot reach 100%, and the protective efficacy of the COVID-19 vaccine ranges from 50.7 to 95.0% (29–39), as shown in Table 4. Due to the limitations of clinical trials, vaccine development, and the fact that any vaccines have been used for <1 year, the protective efficacy of the COVID-19 vaccine is still under continuous observation. Therefore, we assumed that the protective efficacy of the vaccine is the median in Table 4, that was 78.1% (95% *CI:* 64.8–86.3%) (29). So the probability of transmission β_1_, β_2_ among athletes vaccinated fell by 21.9% (95% *CI*: 13.7–35.2%). Variants of COVID-19 can lead to a decrease in neutralizing activity of the vaccine-induced antibodies, especially the mutant strain B.1.351, but the antibodies still possess a certain or high neutralizing ability to the mutant strain (33, 40–70). At present, there is not enough evidence that viral variation has a significant impact on the protection of the new coronavirus vaccine. So, we did not consider the mutations of COVID-19 from different countries. The specific parameter adjustments are shown in Table 5.

**Table 4:** The protective efficacy of vaccines.

**Vaccine type**	**Vaccine name**	**Protective efficacy (%)**	**95% CI**
Inactivated vaccine	SinoVac	78.1	64.8–86.3%
	CoronaVac	50.7	35.9–62.0%
Nucleic acid vaccine	Pfizer mRNA vaccine (BNT162b2)	93.0	78.0–98.0%
	Moderna mRNA vaccine (mRNA-1273)	82.0	20.0–96.0%
Adenovirus vector vaccine	Ad26.COV2.S	66.9	59.0–73.4%
	AstraZeneca	70.4	54.8–80.6%
	Gam-COVID-Vac	87.6	81.1–91.8%

*The protective efficacy in the table indicates the protective efficacy in preventing the occurrence of symptomatic COVID-19*.

**Table 5:** Parameter adjustment and transmission after vaccination.

**Prevention and control measures**	**Parameter adjustment**		**Setting basis/vaccination rate (%)**	**Secondary infectors on the 17th day**	**Peak hour of onset/d**
No intervention (Figure 3)	–		–	587.8	12
Vaccinated but not quarantined. Assume pre-flight screening can identify 20% of asymptomatic infectors (Figure 5)	*r*_1_ = 1.2, *r*_2_ = 1.22, *A*(0) = 8	β_1_ = 0.1083 (0.1031, 0.1167), β_2_ = 0.5414 (0.5156, 0.5833).	40	79.54 (57.39, 131.72)	27
		β_1_ = 0.0837 (0.0759, 0.0962), β_2_ = 0.4184 (0.3797, 0.4812).	60	15.17 (8.52, 36.50)	35
		β_1_ = 0.0591 (0.0488, 0.0758), β_2_ = 0.2954 (0.2438, 0.3792).	80	2.19 (0.87, 8.45)	56
		β_1_ = 0.0468 (0.0352, 0.0656), β_2_ = 0.2339 (0.1758, 0.3282).	90	0.72 (0.23, 3.78)	87
		β_1_ = 0.0406 (0.0284, 0.0605), β_2_ = 0.2032 (0.1418, 0.3027).	95	0.40 (0.11, 2.47)	125
Vaccinated but not quarantined. Assume pre-flight screening can identify 40% of asymptomatic infectors (Figure 6)	*r*_1_ = 1.2, *r*_2_ = 1.22, *A*(0) = 6	β_1_ = 0.1083 (0.1031, 0.1167), β_2_ = 0.5414 (0.5156, 0.5833).	40	60.48 (43.50, 100.85)	27
		β_1_ = 0.0837 (0.0759, 0.0962), β_2_ = 0.4184 (0.3797, 0.4812).	60	11.43 (6.41, 27.58)	36
		β_1_ = 0.0591 (0.0488, 0.0758), β_2_ = 0.2954 (0.2438, 0.3792).	80	1.64 (0.65, 6.36)	58
		β_1_ = 0.0468 (0.0352, 0.0656), β_2_ = 0.2339 (0.1758, 0.3282).	90	0.54 (0.17, 2.84)	90
		β_1_ = 0.0406 (0.0284, 0.0605), β_2_ = 0.2032 (0.1418, 0.3027).	95	0.30 (0.08, 1.86)	130
Vaccination and 7-day compulsory quarantine. Assume pre-flight screening can identify 20% of asymptomatic infectors, 7-day compulsory quarantine has about an 18% risk of missed detection (Figure 7)	*r*_1_ = 1.2, *r*_2_ = 1.22, *A*(0) = 1.44	β_1_ = 0.1083 (0.1031, 0.1167), β_2_ = 0.5414 (0.5156, 0.5833).	40	14.98 (10.70, 25.39)	31
		β_1_ = 0.0837 (0.0759, 0.0962), β_2_ = 0.4184 (0.3797, 0.4812).	60	2.77 (1.55, 6.74)	41
		β_1_ = 0.0591 (0.0488, 0.0758), β_2_ = 0.2954 (0.2438, 0.3792).	80	0.40 (0.16, 1.54)	67
		β_1_ = 0.0468 (0.0352, 0.0656), β_2_ = 0.2339 (0.1758, 0.3282).	90	0.13 (0.04, 0.68)	105
		β_1_ = 0.0406 (0.0284, 0.0605), β_2_ = 0.2032 (0.1418, 0.3027).	95	0.07 (0.02, 0.45)	153
Vaccination and 7-day compulsory quarantine. Assume pre-flight screening can identify 40% of asymptomatic infectors, 7-day compulsory quarantine has about an 18% risk of missed detection (Figure 8)	*r*_1_ = 1.2, *r*_2_ = 1.22, *A*(0) = 1.08	β_1_ = 0.1083 (0.1031, 0.1167), β_2_ = 0.5414 (0.5156, 0.5833).	40	11.27 (8.04, 19.11)	32
		β_1_ = 0.0837 (0.0759, 0.0962), β_2_ = 0.4184 (0.3797, 0.4812).	60	2.08 (1.16, 5.06)	42
		β_1_ = 0.0591 (0.0488, 0.0758), β_2_ = 0.2954 (0.2438, 0.3792).	80	0.30 (0.12, 1.15)	69
		β_1_ = 0.0468 (0.0352, 0.0656), β_2_ = 0.2339 (0.1758, 0.3282).	90	0.10 (0.03, 0.51)	108
		β_1_ = 0.0406 (0.0284, 0.0605), β_2_ = 0.2032 (0.1418, 0.3027).	95	0.05 (0.01, 0.34)	158
Vaccination and 14-day compulsory quarantine. Assume pre-flight screening can identify 20% of asymptomatic infectors, 14-day compulsory quarantine has about a 4% risk of missed detection (Figure 9)	*r*_1_ = 1.2, *r*_2_ = 1.22, *A*(0) = 0.32	β_1_ = 0.1083 (0.1031, 0.1167), β_2_ = 0.5414 (0.5156, 0.5833).	40	3.36 (2.39, 5.71)	35
		β_1_ = 0.0837 (0.0759, 0.0962), β_2_ = 0.4184 (0.3797, 0.4812).	60	0.62 (0.34, 1.50)	46
		β_1_ = 0.0591 (0.0488, 0.0758), β_2_ = 0.2954 (0.2438, 0.3792).	80	0.09 (0.04, 0.34)	76
		β_1_ = 0.0468 (0.0352, 0.0656), β_2_ = 0.2339 (0.1758, 0.3282).	90	0.03 (0.01, 0.15)	120
		β_1_ = 0.0406 (0.0284, 0.0605), β_2_ = 0.2032 (0.1418, 0.3027).	95	0.02 (0, 0.10)	177
Vaccination and 14-day compulsory quarantine. Assume pre-flight screening can identify 40% of asymptomatic infectors, 14-day compulsory quarantine has about a 4% risk of missed detection (Figure 10)	*r*_1_ = 1.2, *r*_2_ = 1.22, *A*(0) = 0.24	β_1_ = 0.1083 (0.1031, 0.1167), β_2_ = 0.5414 (0.5156, 0.5833).	40	2.52 (1.80, 4.29)	36
		β_1_ = 0.0837 (0.0759, 0.0962), β_2_ = 0.4184 (0.3797, 0.4812).	60	0.46 (0.26, 1.13)	47
		β_1_ = 0.0591 (0.0488, 0.0758), β_2_ = 0.2954 (0.2438, 0.3792).	80	0.07 (0.03, 0.26)	78
		β_1_ = 0.0468 (0.0352, 0.0656), β_2_ = 0.2339 (0.1758, 0.3282).	90	0.02 (0.01, 0.11)	123
		β_1_ = 0.0406 (0.0284, 0.0605), β_2_ = 0.2032 (0.1418, 0.3027).	95	0.01 (0, 0.07)	182

*Except “No intervention,” all other situations considered “Keep a minimum of 2 m from athletes at all times. Keep a minimum of 1 m from others. Take two COVID-19 tests before their flight to Japan”*.

It can be found from Figures 5–10 that the assumption of the protective efficacy of the vaccine was 78.1%. Whether isolated or not, screening before take-off can identify 20% or 40% of asymptomatic infectors, which significantly reduced the number of secondary infectors on the 17th day and delayed the peak hour of onset compared to no interventions and current interventions by the JOC. In the comparison of different vaccination rates of various measures, it can be found that when the vaccination rate of athletes reached 80%, the number of secondary infectors on the 17th day decreased significantly. When vaccination rates were 80, 90, and 95%, cumulative infectors still reached a relatively high value at the end, but the first 17 days were very low. It showed that the vaccine was effective, and that extensive vaccination would be useful for the prevention and control of COVID-19 in the Tokyo Olympic Games.

Comparing Figure 5 and Figure 6, Figure 7 and Figure 8, and Figure 9 and Figure 10, it can be seen that under the intervention measures of “vaccinated but not quarantined,” “vaccination and 7-day compulsory quarantine,” and “vaccination and 14-day compulsory quarantine,” the proportion of asymptomatic infected individuals identified by pre-flight screening had little effect on the number of secondarily infected individuals, and the outbreak time was basically the same under different vaccination proportions. However, for the intervention measures in Figures 5–10, as the vaccination rate increased, the secondary infectors on the 17th day and the peak hour of onset significantly decreased. When the vaccination rate of athletes reached 80%, the number of secondary infectors decreased significantly, and it was controlled within 1 when the vaccination rate of athletes reached 90%, indicating that the immune barrier could be established when the vaccination rate reached 80–90%.

Considering that athletes are generally physically fit, we took differences in population resistance into account. We assumed that the protective efficacy of the vaccine of athletes is 15% higher than the average person (78.1%), that is 89.8% (95% *CI*: 74.5–99.2%). Under this assumption, we developed new simulations. The optimal parameters are shown in Table 6.

**Table 6:** Parameter adjustment (Consider differences in population resistance).

**Prevention and control measures**	**Parameter adjustment**		**Setting basis/vaccination rate (%)**	**Secondary infectors on the 17th day**	**Peak hour of onset/d**
No intervention (Figure 3)	–		–	587.8	12
Vaccinated but not quarantined. Assume pre-flight screening can identify 20% of asymptomatic infectors (Figure 11)	*r*_1_ = 1.2, *r*_2_ = 1.22, *A*(0) = 8	β_1_ = 0.1009 (0.0950, 0.1105), β_2_ = 0.5045 (0.4749, 0.5527).	40	49.71 (33.54, 91.36)	29
		β_1_ = 0.0726 (0.0637, 0.0871), β_2_ = 0.3631 (0.3187, 0.4354).	60	6.60 (3.23, 19.38)	42
		β_1_ = 0.0443 (0.0325, 0.0636), β_2_ = 0.2217 (0.1625, 0.3181).	80	0.57 (0.17, 3.20)	99
		β_1_ = 0.0302 (0.0169, 0.0519), β_2_ = 0.1510 (0.0844, 0.2594).	90	0.13 (0.02, 1.16)	9
		β_1_ = 0.0231 (0.0091, 0.0460), β_2_ = 0.1157 (0.0454, 0.2301).	95	0.06 (0.01, 0.67)	7
Vaccinated but not quarantined. Assume pre-flight screening can identify 40% of asymptomatic infectors (Figure 12)	*r*_1_ = 1.2, *r*_2_ = 1.22, *A*(0) = 6	β_1_ = 0.1009 (0.0950, 0.1105), β_2_ = 0.5045 (0.4749, 0.5527).	40	37.64 (25.34, 69.58)	29
		β_1_ = 0.0726 (0.0637, 0.0871), β_2_ = 0.3631 (0.3187, 0.4354).	60	4.96 (2.43, 14.61)	43
		β_1_ = 0.0443 (0.0325, 0.0636), β_2_ = 0.2217 (0.1625, 0.3181).	80	0.43 (0.13, 2.40)	102
		β_1_ = 0.0302 (0.0169, 0.0519), β_2_ = 0.1510 (0.0844, 0.2594).	90	0.10 (0.02, 0.87)	9
		β_1_ = 0.0231 (0.0091, 0.0460), β_2_ = 0.1157 (0.0454, 0.2301).	95	0.04 (0, 0.51)	7
Vaccination and 7-day compulsory quarantine. Assume pre-flight screening can identify 20% of asymptomatic infectors, 7-day compulsory quarantine has about an 18% risk of missed detection (Figure 13)	*r*_1_ = 1.2, *r*_2_ = 1.22, *A*(0) = 1.44	β_1_ = 0.1009 (0.0950, 0.1105), β_2_ = 0.5045 (0.4749, 0.5527).	40	9.24 (6.18, 17.30)	33
		β_1_ = 0.0726 (0.0637, 0.0871), β_2_ = 0.3631 (0.3187, 0.4354).	60	1.20 (0.59, 3.55)	49
		β_1_ = 0.0443 (0.0325, 0.0636), β_2_ = 0.2217 (0.1625, 0.3181).	80	0.10 (0.03, 0.58)	120
		β_1_ = 0.0302 (0.0169, 0.0519), β_2_ = 0.1510 (0.0844, 0.2594).	90	0.02 (0, 0.21)	9
		β_1_ = 0.0231 (0.0091, 0.0460), β_2_ = 0.1157 (0.0454, 0.2301).	95	0.01 (0, 0.12)	7
Vaccination and 7-day compulsory quarantine. Assume pre-flight screening can identify 40% of asymptomatic infectors, 7-day compulsory quarantine has about an 18% risk of missed detection (Figure 14)	*r*_1_ = 1.2, *r*_2_ = 1.22, *A*(0) = 1.08	β_1_ = 0.1009 (0.0950, 0.1105), β_2_ = 0.5045 (0.4749, 0.5527).	40	6.94 (4.64, 13.01)	34
		β_1_ = 0.0726 (0.0637, 0.0871), β_2_ = 0.3631 (0.3187, 0.4354).	60	0.90 (0.44, 2.66)	50
		β_1_ = 0.0443 (0.0325, 0.0636), β_2_ = 0.2217 (0.1625, 0.3181).	80	0.08 (0.02, 0.43)	123
		β_1_ = 0.0302 (0.0169, 0.0519), β_2_ = 0.1510 (0.0844, 0.2594).	90	0.02 (0, 0.16)	9
		β_1_ = 0.0231 (0.0091, 0.0460), β_2_ = 0.1157 (0.0454, 0.2301).	95	<0.01 (0, 0.09)	7
Vaccination and 14-day compulsory quarantine. Assume pre-flight screening can identify 20% of asymptomatic infectors, 14-day compulsory quarantine has about a 4% risk of missed detection (Figure 15)	*r*_1_ = 1.2, *r*_2_ = 1.22, *A*(0) = 0.32	β_1_ = 0.1009 (0.0950, 0.1105), β_2_ = 0.5045 (0.4749, 0.5527).	40	2.06 (1.38, 3.88)	37
		β_1_ = 0.0726 (0.0637, 0.0871), β_2_ = 0.3631 (0.3187, 0.4354).	60	0.27 (0.13, 0.79)	56
		β_1_ = 0.0443 (0.0325, 0.0636), β_2_ = 0.2217 (0.1625, 0.3181).	80	0.02 (0.01, 0.13)	137
		β_1_ = 0.0302 ( 0.0169, 0.0519), β_2_ = 0.1510 (0.0844, 0.2594).	90	<0.01 (0, 0.05)	9
		β_1_ = 0.0231 (0.0091, 0.0460), β_2_ = 0.1157 (0.0454, 0.2301).	95	<0.01 (0, 0.03)	7
Vaccination and 14-day compulsory quarantine. Assume pre-flight screening can identify 40% of asymptomatic infectors, 14-day compulsory quarantine has about a 4% risk of missed detection (Figure 16)	*r*_1_ = 1.2, *r*_2_ = 1.22, *A*(0) = 0.24	β_1_ = 0.1009 (0.0950, 0.1105), β_2_ = 0.5045 (0.4749, 0.5527).	40	1.55 (1.04, 2.91)	38
		β_1_ = 0.0726 (0.0637, 0.0871), β_2_ = 0.3631 (0.3187, 0.4354).	60	0.20 (0.10, 0.59)	57
		β_1_ = 0.0443 (0.0325, 0.0636), β_2_ = 0.2217 (0.1625, 0.3181).	80	0.02 (0, 0.10)	142
		β_1_ = 0.0302 (0.0169, 0.0519), β_2_ = 0.1510 (0.0844, 0.2594).	90	<0.01 (0, 0.04)	9
		β_1_ = 0.0231 (0.0091, 0.0460), β_2_ = 0.1157 (0.0454, 0.2301).	95	<0.01 (0, 0.02)	7

*Except “No intervention,” all other situations considered “Keep a minimum of 2 m from athletes at all times. Keep a minimum of 1 m from others. Take two COVID-19 tests before their flight to Japan”*.

From Figures 11–16, we know that when the vaccination rate of athletes reached 80%, the number of secondary infectors on the 17th day decreased significantly and was controlled within 1, indicating that the immune barrier could be established when the vaccination rate reached 80%.

## Discussion

The strengths of our study were that the topic is timely, and we used an epidemic method to model the spread of COVID-19. We also discussed the sensitivity of parameters. And we discussed the relationship between vaccination services and spread of COVID-19, considering different protective efficacy of vaccines and vaccination rates. We took differences in population resistance into account as athletes are generally more physically fit. We tested the robustness of the SEIARH model and found that the model was stable. The model we constructed considered the time, environment, and behavior at the same time. The limitations were that we did not consider the transmission of COVID-19 from athletes to the audience, because the policy of JOC clearly limited the scope of activities of athletes and the distance from the audience (6, 7, 71). But we considered the latest results of protective efficacy of the vaccine to make the results more scientific and credible (28, 29). There was no specific number of participants from each country, so it was not possible to accurately estimate the initially infected individuals. The parameters of the model refer to other research based on real data, which may be different from the real situation in Japan, but we set the interval of parameters to reduce bias.

Through the simulation of no intervention measures and various intervention measures, we found that the prevention and control effect of the measures currently proposed by JOC was not ideal. The total number of secondary infectors could still reach the level of no intervention measures, but with a longer duration. However, the prevention and control effect of the intervention measures proposed by us was significantly better than that of no intervention measures or the epidemic response measures proposed by JOC. The simulated number of secondary infectors without intervention measures on the 17th day was 587.8. As shown in Tables 3, 5, 6, we simulated no interventions and different levels of intervention. It showed that these measures can delay the outbreak of COVID-19 to some extent, but the number of infectors was not well-controlled. If the protective efficacy of the vaccine was 78.1% and the vaccination rate reached 90%, the number of secondary infectors under all intervention measures was <1; if the protective efficacy of vaccine was 89.8% and the vaccination rate reached 80%, the number of secondary infectors under all intervention measures was <1, indicating that the epidemic situation was effectively controlled, and vaccination can effectively prevent COVID-19. Comparing Table 5 and Table 6, it was found that under the same intervention circumstance, if the protective efficacy of vaccine was 89.8%, the number of secondary infectors decreased significantly. But the peak hour of onset for each control measure in Table 5 kept being postponed, while it was postponed first and then advanced in Table 6. This was because when the protective efficacy of vaccine was 89.8%, it could not trigger the transmission of COVID-19 and the transmission period was short. Vaccines gave a protection ability to COVID-19 infectors. Studies have found that vaccine uptake was the “main driver” of the decline in COVID-19 infectors rather than lockdown (72). Although there are variant strains of COVID-19, the vaccines still possessed a neutralizing ability to the mutant strain (33, 40–70). Both the Oxford AstraZeneca and Pfizer BioNTech COVID-19 vaccines were effective in reducing the risk of SARS-CoV-2 infection and COVID-19 hospitalization in people with the Delta Variant (55). Because people who participate in the Tokyo Olympic Games will have closed contact (including athletes, staff, etc.), and the vaccine supply can also be guaranteed, we recommend that they should be vaccinated.

As of June 10, 2021, only 4.3% of Japan's population of about 126 million was fully vaccinated against COVID-19, and only 12.6% of the total population was partly vaccinated against COVID-19 (73). At the same time, the Japanese government is trying to complete the task of vaccinating one million times a day, so as to vaccinate all 36 million elderly people in the country before the Tokyo Olympic Games (74). So the prevention and control measures against COVID-19 in the Tokyo Olympic Games need to be further strengthened. In the face of infectious diseases such as COVID-19 with strong infectivity, in addition to the prevention and control measures listed in this study, the combination of multiple intervention measures will bring better results. For example, closed-loop management can be adopted during the Olympic Games, sites can be disinfected before the athletes arrive, and the staff can be trained on epidemic prevention and control.

Risk assessment includes risk identification, dose-response relationship, exposure assessment, etc. COVID-19 is mainly transmitted through direct transmission, aerosol transmission, and contact transmission. Therefore, we started from the potential risks, namely, intervening in the contact between people, then simulated the potential transmission risks, and finally formed a risk assessment for COVID-19. In this study, a simulation method of public health intervention prevention and control based on the dynamic model of COVID-19 was proposed, which can be extended to all kinds of large-scale activities or infectious disease prevention and control research. The intervention measures in this study were based on the epidemic prevention and control measures and ideas proposed by JOC and the IOC. At present, many countries can produce the COVID-19 vaccine, and IOC will also bear the cost of the vaccine, so it is feasible (8). Vaccination is not compulsory, so it does not involve human rights interventions, and it is a complex regulation. People who have been vaccinated still run the risk of infection, and there are variant strains, i.e., Alpha, Beta, Gamma, Delta, etc. (75). Therefore, we could consider reclassifying the population (e.g., adding vaccinated and unvaccinated, or/and according to COVID-19 virus subtype) and combining the latest data on the protective efficacy of vaccines in the future, with the purpose of exploring effective prevention and control measures for COVID-19.

COVID-19 has an incubation period, and there were studies showing that the median incubation period was 4 days (interquartile range, 2–7) (76, 77). So athletes may be infectious after returning, and countries need to take relevant prevention and control measures for athletes, such as nucleic acid detection, isolation, etc.

## Conclusions

Based on the dynamic model, this paper simulated different prevention and control measures of the Tokyo Olympic Games, comparing the number of secondary infectors under different measures, and found that vaccination had the best prevention and control effect. When the protective efficacy of the vaccine was 78.1 or 89.8% and if the vaccination rate reached 90%, then the number of secondary infectors can be controlled within 1. Our study will contribute to the formulation of relevant measures by JOC and IOC. In summary, compared with the current public health interventions, mass vaccination will become a milestone in the control of COVID-19.

## Data Availability Statement

The original contributions presented in the study are included in the article/Supplementary Material, further inquiries can be directed to the corresponding author/s.

## Author Contributions

WZ consulted the literature, analyzed the data, wrote the programs, and was a major contributor in writing the manuscript. JF wrote part of the manuscript. CL collected the parameters of the model. HW made comments on the content of the article. YZ collected part of the data. LZ gave advice on setting parameters. XZ made constructive comments on the manuscript. TZ contributed significantly to analysis and manuscript preparation. All authors contributed to the article and approved the submitted version.

## Funding

This research work was funded by Sichuan Science and Technology Program (Grant nos. 2020YFS0015, 2020YFS0091, and 2021YFS0001-LH), Health Commission of Sichuan province (Grant no. 20PJ092), National Natural Science Foundation of China (Grant nos. 81602935 and 82041033), Chongqing Science and Technology Program (Grant no. cstc2020jscx-cylhX0003), Central government funding items (Grant no. 2021zc02), and Liangshan Yi Autonomous Prefecture Center for Disease Control and Prevention (Grant no. H210322). The funders played no role in the design of the study and collection, analysis, and interpretation of data and in writing the manuscript.

## Conflict of Interest

The authors declare that the research was conducted in the absence of any commercial or financial relationships that could be construed as a potential conflict of interest.

## Publisher's Note

All claims expressed in this article are solely those of the authors and do not necessarily represent those of their affiliated organizations, or those of the publisher, the editors and the reviewers. Any product that may be evaluated in this article, or claim that may be made by its manufacturer, is not guaranteed or endorsed by the publisher.

## Supplementary Material

Supplementary Material A Introduces a way to estimate parameters by data-driven methods.

Supplementary Material B Shares our codes.

## Abbreviations

COVID-19: coronavirus disease 2019
SEIARH: susceptible-exposed-symptomatic-asymptomatic-recovered-hospitalized
SEIR: susceptible-exposed-infectious-recovered
IOC: International Olympic Committee
JOC: Japan Olympic Committee.
