# Supplementary material for: COVID-19 Risk Assessment for the Tokyo Olympic Games
Source: Front Public Health. 2021 Oct 25;9:730611. doi: 10.3389/fpubh.2021.730611 (PMC8572808; doi:10.3389/fpubh.2021.730611)
Supplement: Supplementary Material A — Introduces a way to estimate parameters by data-driven methods. [file Data_Sheet_1.doc]

Supplementary Material A

**Parameters Estimation of SIR Model based on MCMC**

The period from January to March in 2020 is the most serious period of COVID -19 in Wuhan, so we used the relevant data of COVID-19 in Wuhan from January 11 to February 13 in 2020 to estimate the parameters of the model based on Markov Chain Monte Carlo Method (MCMC). Due to the limitation of the public data, we only estimated the propagation parameters between Infectious (I) and Recovered (R) in susceptible-infectious-recovered (SIR) model, which is “recovery rate of symptomatic infected individuals”.

# 1. Data source

The data collected by R package “ncov2019” comes from the public data of COVID-19 epidemic situation of the National Health Commission of the People’s Republic of China [1], which includes daily infected number (I), recovered number (R) and death number (D), with no missing value, abnormal value and high reliability. Therefore, this paper obtained the historical data of Wuhan COVID-19 from January 11 to February 13, 2020 based on R package “ncov2019”, as shown in **Figure. 1**.

Figure. 1 Historical data of Wuhan COVID-19

# 2. Methods

Due to the limitation of data, this paper only estimates the propagation probability between I and R, as shown in **Figure. 2**. The transition of an individual from one state to another can be regarded as a random process, and the time length of an individual in a certain interval follows exponential distribution. If the parameter of exponential distribution is $\lambda$, the probability of the individual leaving the current state in the time interval is $1-e^{-\lambda h}$. In addition, the number of people flowing into a certain state in a day can be generated by binomial distribution. The number of experiments in the binomial distribution is the number of individuals in the current interval [2].


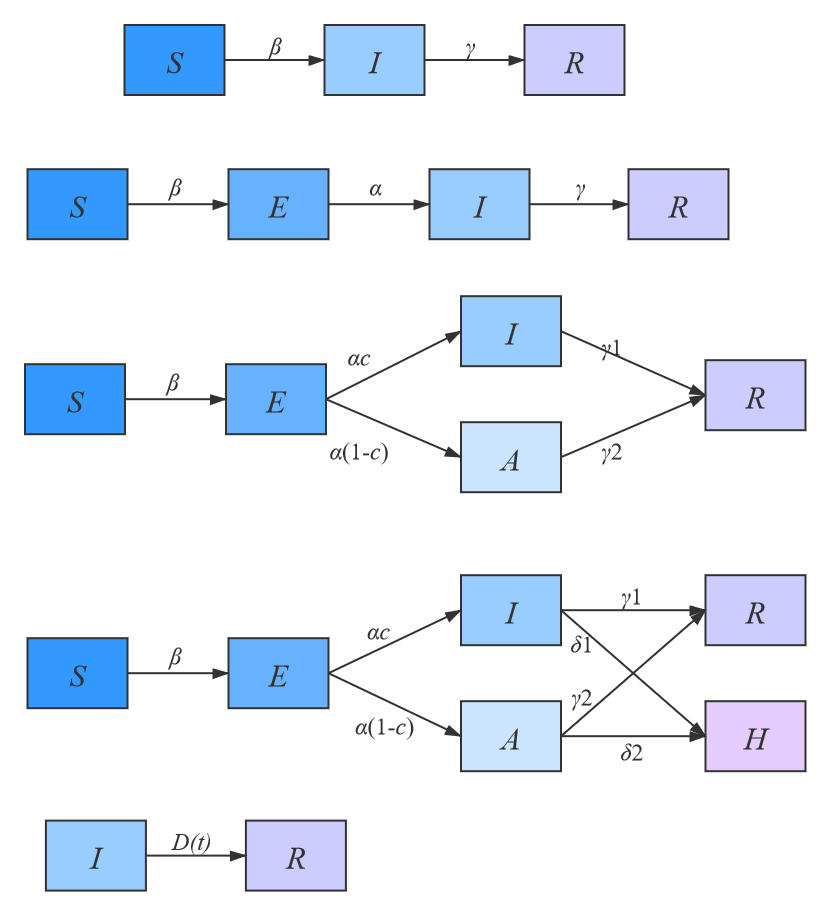


Figure. 2 Flow diagram of parameter estimation

$D(t)$ in **Figure. 2** shows the number of people transferred from I to R, which follows binomial distribution, as $D(t)\sim Bin\left(I\left(t\right),P_{R}\right)$, The calculation formula of $P_{R}$ is as follows:

| $P_{R}=1-e^{-\gamma h}$, | (1) |
| --- | --- |

where $h$ is the time interval; $\gamma$ is the propagation probability between I and R.

Parameter $\gamma$ has its independent gamma prior [3], as $\gamma\sim Gamma\left(a,b\right)$. Its mean is $a/b$, and the variance is $a/b^{2}$. *b* is the number of previous observations, *a* is the sum of the previously observed counts of *b* [4]. And in this study, *a* refers to the current number, *b* refers to the total number. Then the prior distribution of parameter $\gamma$ is as follows:

| $f(\gamma)=\left\{\begin{matrix} \frac{N^{I(t)}\gamma^{I(t)-1}e^{-N\lambda}}{\tau\left(I(t)\right)}\text{ },\gamma\in[0,1] \\ 0\text{ },else \end{matrix}\right.$, | (2) |
| --- | --- |

where $N$ is the total population number.

We used MCMC for parameter estimation, a likelihood function was constructed based on existing data, the formula is as follows:

| $L\left(D\vert\gamma\right)=\prod_{t} \left(1-e^{-\gamma h}\right)^{z(t)}\cdot\left(e^{-\gamma h}\right)^{I(0)-z(t)}$, | (3) |
| --- | --- |

where $z(t)$ is the number of people increased at the current time; $I(0)$ is the number of infected people at the initial time.

# 3. Results

Using software to solve equation (3), the results are shown in **Figure. 3**. By observing $\gamma$, it can be seen that MCMC estimation of parameters is convergent. The mean value of $\gamma$ is 0.0085, 95% *CI* is (0.0085, 0.0085). According to the data of COVID-19 epidemic in Wuhan from January 11 to February 13, 2020, the result of 0.0085 is equal to He [2].

Figure. 3 Parameter $\gamma$ trajectories

# Reference

1. National Health Commission of the People’s Republic of China. Coronavirus statistics reports of the world (2021). https://news.qq.com/zt2020/page/feiyan.htm. [Accessed July 1 2021].
2. He S, Tang S Y, Rong L. A discrete stochastic model of the COVID-19 outbreak: Forecast and control. Mathematical Biosciences and Engineering (2020) 17(4): 2792-2804. doi: 10.3934/mbe.2020153
3. Lekone P E, Finkenstädt B F. Statistical Inference in a Stochastic Epidemic SEIR Model with Control Intervention: Ebola as a Case Study. Biometrics (2006) 62(4): 1170-1177. doi: 10.1111/j.1541-0420.2006.00609.x
4. Hoff P D. A First Course in Bayesian Statistical Methods. New York: Springer (2009). doi: 10.1007/978-0-387-92407-6
