# Supplementary material for: COVID-19 Risk Assessment for the Tokyo Olympic Games
Source: Front Public Health. 2021 Oct 25;9:730611. doi: 10.3389/fpubh.2021.730611 (PMC8572808; doi:10.3389/fpubh.2021.730611)
Supplement: Supplementary Material B — Shares our codes. [file Data_Sheet_2.doc]

Supplementary Material B

**Code**

Because the codes are long and similar, we only list the codes in **Figure. 5**. Details are as follows:


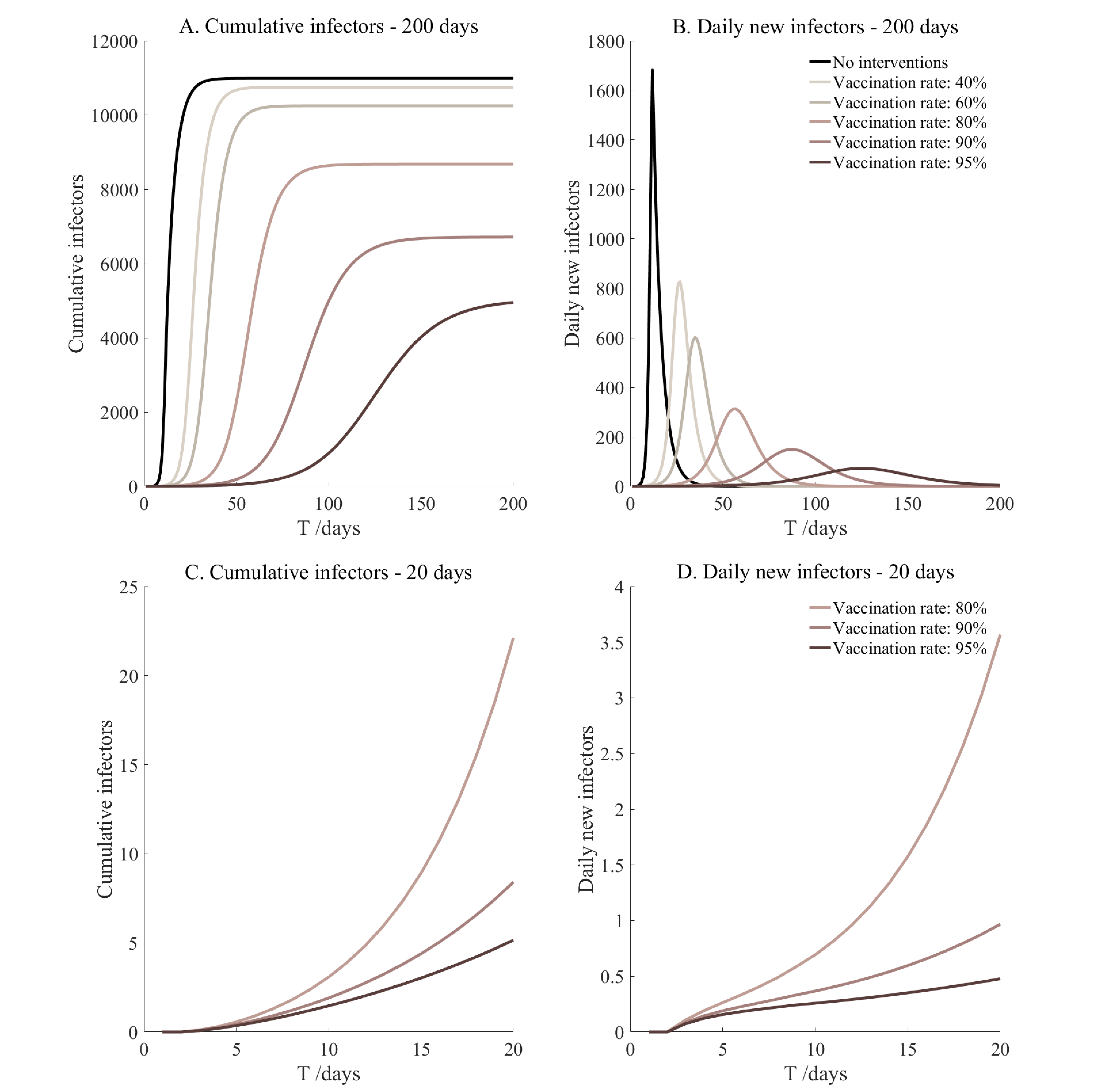


Figure. 5 Vaccinated but not quarantined. Assume pre-flight screening can identify 20% of asymptomatic infectors

| clear  clc  close all  %% Parameter setting without intervention  N = 1.1*10^4;  E = 0;  I = 0;  A = 10;  S = N - I - A;  R = 0;  H = 0;  r1 = 2.20;  r2 = 2.22;  beta1 = 0.15747;  beta2 = 0.78735;  alpha = 1/5.2;  c=0.4;  gamma1 = 0.0085;  gamma2 = 0.0085;  gamma3 = 0.15;  delta1 = 0.60;  delta2 = 0.40;  T = 1:200;  %% no intervention  for idx = 1:length(T)-1  if idx>=7  gamma1=1;  gamma2=0.5;  end  S(idx+1)=S(idx) - r1*beta1*S(idx)*(I(idx)+A(idx))/N - r2*beta2*S(idx)*E(idx)/N;  E(idx+1)=E(idx) + r1*beta1*S(idx)*(I(idx)+A(idx))/N -alpha*E(idx) + r2*beta2*S(idx)*E(idx)/N;  I(idx+1)=I(idx) + alpha*c*E(idx) - gamma1*I(idx) - delta1*I(idx);  A(idx+1)=A(idx) + alpha*(1-c)*E(idx) - gamma2*A(idx) - delta2*A(idx);  H(idx+1)=H(idx) + delta1*I(idx) + delta2*A(idx) - gamma3*H(idx);  R(idx+1)=R(idx) + gamma1*I(idx) + gamma2*A(idx) + gamma3*H(idx);  leiji1(idx+1)=alpha*E(idx);  new1(idx+1)=alpha*E(idx);  end  %% cumulative number without intervention  II=I+A;  for i = 1:length(T)-1  leiji1(i+1)=leiji1(i)+leiji1(i+1);  end  %% 40%  A2 = 8;  S = N - I - A2;  E = 0; I = 0; R = 0; H = 0;  gamma1 = 0.0085;  gamma2 = 0.0085;  beta1=0.1083;  beta2=0.5414;  for idx = 1:length(T)-1  if idx>=1  r1 = 1.2;  r2 = 1.22;  end  if idx>=7  gamma1=1;  gamma2=0.5;  end  S(idx+1)=S(idx) - r1*beta1*S(idx)*(I(idx)+A2(idx))/N - r2*beta2*S(idx)*E(idx)/N;  E(idx+1)=E(idx) + r1*beta1*S(idx)*(I(idx)+A2(idx))/N -alpha*E(idx) + r2*beta2*S(idx)*E(idx)/N;  I(idx+1)=I(idx) + alpha*c*E(idx) - gamma1*I(idx) - delta1*I(idx);  A2(idx+1)=A2(idx) + alpha*(1-c)*E(idx) - gamma2*A2(idx) - delta2*A2(idx);  H(idx+1)=H(idx) + delta1*I(idx) + delta2*A2(idx) - gamma3*H(idx);  R(idx+1)=R(idx) + gamma1*I(idx) + gamma2*A2(idx) + gamma3*H(idx);  leiji2(idx+1)=alpha*E(idx);  new2(idx+1)=alpha*E(idx);  end  for i = 1:length(T)-1  leiji2(i+1)=leiji2(i)+leiji2(i+1);  end  %% 60%  A3 = 8;  S = N - I - A3;  E = 0; I = 0; R = 0; H = 0;  gamma1 = 0.0085;  gamma2 = 0.0085;  beta1=0.0837;  beta2=0.4184;  for idx = 1:length(T)-1  if idx>=1  r1 = 1.2;  r2 = 1.22;  end  if idx>=7  gamma1=1;  gamma2=0.5;  end  S(idx+1)=S(idx) - r1*beta1*S(idx)*(I(idx)+A3(idx))/N - r2*beta2*S(idx)*E(idx)/N;  E(idx+1)=E(idx) + r1*beta1*S(idx)*(I(idx)+A3(idx))/N -alpha*E(idx) + r2*beta2*S(idx)*E(idx)/N;  I(idx+1)=I(idx) + alpha*c*E(idx) - gamma1*I(idx) - delta1*I(idx);  A3(idx+1)=A3(idx) + alpha*(1-c)*E(idx) - gamma2*A3(idx) - delta2*A3(idx);  H(idx+1)=H(idx) + delta1*I(idx) + delta2*A3(idx) - gamma3*H(idx);  R(idx+1)=R(idx) + gamma1*I(idx) + gamma2*A3(idx) + gamma3*H(idx);  leiji3(idx+1)=alpha*E(idx);  new3(idx+1)=alpha*E(idx);  end  for i = 1:length(T)-1  leiji3(i+1)=leiji3(i)+leiji3(i+1);  end  %% 80%  A2 = 8;  S = N - I - A2;  E = 0; I = 0; R = 0; H = 0;  gamma1 = 0.0085;  gamma2 = 0.0085;  beta1=0.0591;  beta2=0.2954;  for idx = 1:length(T)-1  if idx>=1  r1 = 1.2;  r2 = 1.22;  end  if idx>=7  gamma1=1;  gamma2=0.5;  end  S(idx+1)=S(idx) - r1*beta1*S(idx)*(I(idx)+A2(idx))/N - r2*beta2*S(idx)*E(idx)/N;  E(idx+1)=E(idx) + r1*beta1*S(idx)*(I(idx)+A2(idx))/N -alpha*E(idx) + r2*beta2*S(idx)*E(idx)/N;  I(idx+1)=I(idx) + alpha*c*E(idx) - gamma1*I(idx) - delta1*I(idx);  A2(idx+1)=A2(idx) + alpha*(1-c)*E(idx) - gamma2*A2(idx) - delta2*A2(idx);  H(idx+1)=H(idx) + delta1*I(idx) + delta2*A2(idx) - gamma3*H(idx);  R(idx+1)=R(idx) + gamma1*I(idx) + gamma2*A2(idx) + gamma3*H(idx);  leiji4(idx+1)=alpha*E(idx);  new4(idx+1)=alpha*E(idx);  end  for i = 1:length(T)-1  leiji4(i+1)=leiji4(i)+leiji4(i+1);  end  %% 90%  A2 = 8;  S = N - I - A2;  E = 0; I = 0; R = 0; H = 0;  gamma1 = 0.0085;  gamma2 = 0.0085;  beta1=0.0468;  beta2=0.2339;  for idx = 1:length(T)-1  if idx>=1  r1 = 1.2;  r2 = 1.22;  end  if idx>=7  gamma1=1;  gamma2=0.5;  end  S(idx+1)=S(idx) - r1*beta1*S(idx)*(I(idx)+A2(idx))/N - r2*beta2*S(idx)*E(idx)/N;  E(idx+1)=E(idx) + r1*beta1*S(idx)*(I(idx)+A2(idx))/N -alpha*E(idx) + r2*beta2*S(idx)*E(idx)/N;  I(idx+1)=I(idx) + alpha*c*E(idx) - gamma1*I(idx) - delta1*I(idx);  A2(idx+1)=A2(idx) + alpha*(1-c)*E(idx) - gamma2*A2(idx) - delta2*A2(idx);  H(idx+1)=H(idx) + delta1*I(idx) + delta2*A2(idx) - gamma3*H(idx);  R(idx+1)=R(idx) + gamma1*I(idx) + gamma2*A2(idx) + gamma3*H(idx);  leiji5(idx+1)=alpha*E(idx);  new5(idx+1)=alpha*E(idx);  end  for i = 1:length(T)-1  leiji5(i+1)=leiji5(i)+leiji5(i+1);  end  %% 95%  A2 = 8;  S = N - I - A2;  E = 0; I = 0; R = 0; H = 0;  gamma1 = 0.0085;  gamma2 = 0.0085;  beta1=0.0406;  beta2=0.2032;  for idx = 1:length(T)-1  if idx>=1  r1 = 1.2;  r2 = 1.22;  end  if idx>=7  gamma1=1;  gamma2=0.5;  end  S(idx+1)=S(idx) - r1*beta1*S(idx)*(I(idx)+A2(idx))/N - r2*beta2*S(idx)*E(idx)/N;  E(idx+1)=E(idx) + r1*beta1*S(idx)*(I(idx)+A2(idx))/N -alpha*E(idx) + r2*beta2*S(idx)*E(idx)/N;  I(idx+1)=I(idx) + alpha*c*E(idx) - gamma1*I(idx) - delta1*I(idx);  A2(idx+1)=A2(idx) + alpha*(1-c)*E(idx) - gamma2*A2(idx) - delta2*A2(idx);  H(idx+1)=H(idx) + delta1*I(idx) + delta2*A2(idx) - gamma3*H(idx);  R(idx+1)=R(idx) + gamma1*I(idx) + gamma2*A2(idx) + gamma3*H(idx);  leiji6(idx+1)=alpha*E(idx);  new6(idx+1)=alpha*E(idx);  end  for i = 1:length(T)-1  leiji6(i+1)=leiji6(i)+leiji6(i+1);  end  %% plot  subplot(1,2,1);  plot(T,leiji1,'k','LineWidth',3);hold on;  plot(T,leiji2,'Color',[217 208 197]/255,'LineWidth',3);hold on;  plot(T,leiji3,'Color',[191 182 170]/255,'LineWidth',3);hold on;  plot(T,leiji4,'Color',[191 157 149]/255,'LineWidth',3);hold on;  plot(T,leiji5,'Color',[166 128 124]/255,'LineWidth',3);hold on;  plot(T,leiji6,'Color',[89 61 59]/255,'LineWidth',3);hold on;  set(gca,'box','off');  set(gca,'FontName','Times New Roman','FontSize',20);  xlabel('T /days','FontName','Times New Roman');ylabel('Cumulative infectors','FontName','Times New Roman')  title('A. Cumulative infectors - 200 days','FontName','Times New Roman');  subplot(1,2,2);  plot(T,new1,'k','LineWidth',3);hold on;  plot(T,new2,'Color',[217 208 197]/255,'LineWidth',3);hold on;  plot(T,new3,'Color',[191 182 170]/255,'LineWidth',3);hold on;  plot(T,new4,'Color',[191 157 149]/255,'LineWidth',3);hold on;  plot(T,new5,'Color',[166 128 124]/255,'LineWidth',3);hold on;  plot(T,new6,'Color',[89 61 59]/255,'LineWidth',3);hold on;  set(gca,'box','off');  set(gca,'FontName','Times New Roman','FontSize',20);  title('B. Daily new infectors - 200 days','FontName','Times New Roman');  xlabel('T /days','FontName','Times New Roman');ylabel('Daily new infectors','FontName','Times New Roman')  z=legend('No interventions','Vaccination rate: 40%','Vaccination rate: 60%','Vaccination rate: 80%','Vaccination rate: 90%','Vaccination rate: 95%');set(z,'FontName','Times New Roman', 'Box', 'off')  %% subplot  figure;  subplot(1,2,1);  plot(T,leiji4,'Color',[191 157 149]/255,'LineWidth',3);hold on;  plot(T,leiji5,'Color',[166 128 124]/255,'LineWidth',3);hold on;  plot(T,leiji6,'Color',[89 61 59]/255,'LineWidth',3);hold on;  set(gca,'box','off');  set(gca,'FontName','Times New Roman','FontSize',20);  xlabel('T /days','FontName','Times New Roman');ylabel('Cumulative infectors','FontName','Times New Roman')  title('C. Cumulative infectors - 20 days','FontName','Times New Roman');  subplot(1,2,2);  plot(T,new4,'Color',[191 157 149]/255,'LineWidth',3);hold on;  plot(T,new5,'Color',[166 128 124]/255,'LineWidth',3);hold on;  plot(T,new6,'Color',[89 61 59]/255,'LineWidth',3);hold on;  set(gca,'box','off');  set(gca,'FontName','Times New Roman','FontSize',20);  title('D. Daily new infectors - 20 days','FontName','Times New Roman');  xlabel('T /days','FontName','Times New Roman');ylabel('Daily new infectors','FontName','Times New Roman')  z=legend('Vaccination rate: 80%','Vaccination rate: 90%','Vaccination rate: 95%');set(z,'FontName','Times New Roman', 'Box', 'off') |
| --- |
